# Dynamics and Energy Relaxation of Formic Acid Dimers Following Inner Valence Ionization

**DOI:** 10.1021/acs.jpclett.6c03064

**Published:** 2026-10-05

**Authors:** J. X. Zhong Manis, Y. S. Wang, M.C. Rohan, S. Kumar, M. Shaikh, J. Stindl, G. Kastirke, O. D. McGinnis, M. Kircher, C. Bagdia, N. Iwamoto, R. Enoki, V. Davis, J. B. Williams, R. Dörner, I. Ben-Itzhak, D. S. Slaughter, T. Weber, J. S. Kretchmer, T. M. Orlando

**Affiliations:** † School of Chemistry and Biochemistry, 1372 Georgia Institute of Technology, Atlanta, Georgia 30332, United States; ‡ Department of Chemistry, 34886 Tamkang University, Tamsui, New Taipei City 251301, Taiwan; ¶ Chemical Sciences Division, Lawrence Berkeley National Laboratory, Berkeley, California 94720, United States; § Institut für Kernphysik, 119482 Goethe-Universität, 60438 Frankfurt am Main, Germany; ∥ J. R. Macdonald Laboratory, Department of Physics, 1666 Kansas State University, Manhattan, Kansas 66506, United States; ⊥ Department of Physics, 9173 University of Nevada, Reno, Nevada 89557, United States; # School of Physics, 54239 Georgia Institute of Technology, Atlanta, Georgia 30332, United States

## Abstract

We study gas-phase dissociation of the formic acid dimer dication as a model for DNA base-pair interactions after inner-valence 56 eV extreme ultraviolet (EUV) ionization. Employing reaction microscopy, we measure the 3D momenta of electrons and fragment-ions in coincidence to reconstruct channel-resolved electron kinetic energies, fragment kinetic energy release, and angular distributions. This work focuses on the symmetric 2-body (HCOOH^+^ + HCOOH^+^) and asymmetric 3-body (HCOOH^+^ + HCOO^+^ + H) breakup channels, reporting their relative yields and dynamics. Electron-energy signatures and angular correlations distinguish direct double ionization (DDI) from sequential ionization (SI) with a ratio of ∼9:5. In SI, low-energy electrons and their near-isotropic angular distributions indicate a competition between local intramolecular autoionization (AI) and nonlocal intermolecular Coulombic decay (ICD). Real-time simulations of the electron dynamics support AI/ICD-driven secondary ionization on sub-20 fs time scales. The SI process depends on whether the initially ionized orbital has carbonyl- or hydroxyl-oxygen character, with charge and energy transfer occurring between diagonally adjacent oxygen atoms rather than along hydrogen bonds.

## Graphical abstract

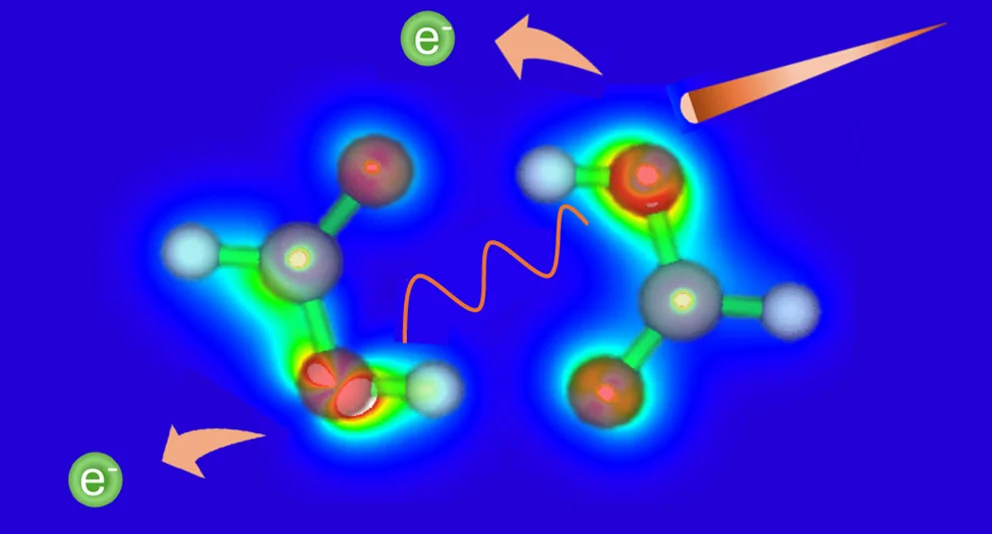

High-energy ionizing radiation, including γ-rays, β-particles (high energy electrons or positrons), swift heavy ions, and X-rays, can induce ionization-driven molecular damage. In complex biomolecular systems like RNA and DNA, ionization and subsequent decay processes can produce single- or double-strand breaks, where the latter is particularly harmful due to the potential for frame-shift mutations in the coding region.^1^ Swift heavy ions induce dense electronic excitation and ionization in biological matter, including core–shell ionization and secondary electron cascades that can drive localized molecular fragmentation and DNA damage.^2^ Similarly, electron and X-ray irradiation damage nucleobases, nucleotides, RNA, and DNA through a combination of direct core ionization and indirect valence ionization pathways.^3–5^

For inner-valence ionization, two primary double-ionization mechanisms can occur: direct double ionization (DDI), in which two electrons are ejected simultaneously following photon absorption, and sequential ionization (SI), in which the molecule is singly ionized and a subsequent electronic relaxation event leads to the emission of a secondary electron. In both cases, the two emitted electrons can come from either the same atomic site or from different atomic sites within a molecule or cluster, and the ionization events can give rise to various nuclear fragmentation channels.^6^ Additionally, in SI, nuclear dynamics can occur before the second electron is emitted, and therefore alter or compete with the secondary ionization process, as, e.g., seen in proton-transfer-mediated charge separation in the water dimer.^7–10^

One key electron relaxation pathway of interest is intermolecular Coulombic decay (ICD), which has attracted considerable attention due to its high efficiency and its role as a prolific source of low energy electrons (LEEs).^10–16^ Briefly, ICD involves ionization of the deep valence or core levels of a molecule. The outer valence shell then decays internally to fill the hole created by the initial ionization, releasing enough energy to ionize the valence level of a neighboring molecule via virtual photon exchange. The result is two positively charged ions in close proximity that subsequently undergo a Coulomb explosion. The ICD process also produces LEEs, which can scatter inelastically, breaking bonds via the formation of transient negative ion resonances. As such, ICD likely plays a key role in radiation-driven charge migration and fragmentation.^11,17,18^

Formic acid (HCOOH) is a simple organic acid involved in several biochemical processes and is frequently considered a precursor to larger biomolecules.^19–22^ Dimerization yields (HCOOH)_2_, which is an appealing model for DNA base-pair interactions due to its double hydrogen bond structure analogous to adenine-thymine pairing.^19,23,24^ Consequently, (HCOOH)_2_ presents a compelling model system for DNA base pairs upon inner valence-shell ionization. Many possible relaxation and breakup pathways yielding two cationic fragments are viable in (HCOOH)_2_. These include autoionization (AI), ICD, proton transfer, neutral hydrogen transfer, and combinations thereof. Several relevant pathways for the symmetric 2-body and asymmetric 3-body fragmentation channels considered here are summarized in Scheme S1 in the Supporting Information. In this work, we focus primarily on assessing the contributions of AI and ICD to two prominent fragmentation channels of (HCOOH)_2_ after initial ionization. We also aim to uncover the associated time scales and mechanisms during electronic relaxation. These results represent an initial step toward a more comprehensive understanding of the production of LEEs in hydrogen-bonded biomolecular dimers.

The electron-impact ionization-driven dissociation of the gas-phase (HCOOH)_2_ cation has been previously studied using fragment-ion and electron coincidence measurements coupled with static electronic structure calculations and ab initio molecular dynamics simulations. In these investigations, the symmetric 2-body channel was attributed primarily to ICD, whereas the asymmetric 3-body channels were assigned to one-site double ionization followed by intermolecular charge transfer (CT).^25,26^ Such one-site dications can arise in photoionization through direct double photoionization or single ionization followed by secondary AI, which makes CT relevant also in the case of AI.^26^

Additionally, the combination of EUV/IR pump–probe and Non-Adiabatic Ab Initio Molecular Dynamics (NA-AIMD) calculations revealed complex hydrogen-bonded proton transfer dynamics on the ground-state cationic surface, which included both ultrafast proton transfer within hundreds of femtoseconds and ultraslow dissociation on the microsecond time scale.^27^ Moreover, the same team showed that double ionization of the formic-acid dimer produces excited-state-driven fragmentation in which nonadiabatic decay after dimer separation triggers roaming of an OH fragment in the presence of a strong NIR field (∼1 × 10^12^ W/cm^2^), enabling both H_2_O^+^ formation and neutral OH ejection.^28^

Here, we investigate the fragmentation dynamics of (HCOOH)_2_ upon EUV ionization using Cold Target Recoil-Ion Momentum Spectroscopy (COLTRIMS; further details can be found in the Methods section).^29–31^ The experimental technique involves the acquisition of 3-D electron and fragment-ion momenta, which are measured in coincidence on a shot-by-shot basis. From these observables, the electron energy (eKE), the fragment kinetic energy release (KER), and the electron and ion angular distributions relative to the EUV polarization direction are then deduced. The measurements are complemented with the first explicit real-time simulation of the correlated electron and hole dynamics in such a complex biomolecular dimer target. These simulations utilize our recently developed non-Hermitian extension of real-time time-dependent density functional theory (RT-TDDFT).^32^ Both experimental and theoretical findings suggest that DDI is the dominant pathway resulting in the prompt expulsion of two electrons and two cationic fragments. Intriguingly, a competitive SI pathway also occurs on a femtosecond time scale, which involves a balance between nonlocal ionization, namely ICD, and local AI, depending on the initial ionization condition.^33–40^

In the present experimental scheme, (HCOOH)_2_ is doubly ionized upon absorption of a single 56 eV linearly polarized synchrotron EUV photon. The reaction channels we focus on in this investigation are

1 $${\left(\mathrm{HCOOH}\right)}_{2}+h\nu \to 2{\mathrm{HCOOH}}^{+}+2e^{-}$$

2 $${\left(\mathrm{HCOOH}\right)}_{2}+h\nu \to {\mathrm{HCOOH}}^{+}+{\mathrm{HCOO}}^{+}+H+2e^{-}$$

where eqs 1 and 2 correspond to the symmetric and asymmetric breakup channels, respectively.

These fragmentation channels can be identified and isolated in the ion Time-of-Flight (tof) correlation map, also known as the photoion–photoion coincidence (PIPICO) spectrum, shown in Figure 1. Due to momentum conservation, ions originating from the same dimer exhibit correlated trajectories, mapping to TOFs along the diagonal lines. Applying COLTRIMS, we isolated the symmetric (eq 1, orange dashed line) and the asymmetric (eq 2, green dotted line) breakup channels in the PIPICO spectrum (see Figure 1). Besides the symmetric 2-body and asymmetric 3-body breakup channels of interest here, the PIPICO spectrum reveals contributions from the 4-body breakup HCOO^+^ + HCOO^+^ + 2H and the 5-body breakup CO_2_^+^ + HCOO^+^ + 3H, indicated by the solid orange and dashed gray lines, respectively. The undetected neutral fragments could alternatively be H_2_ and H_2_ + H, respectively, although these pathways are likely less probable. These contributions partially overlap with the asymmetric 3-body fragmentation channel [see also the sum of the ion TOFs in Figure S2­(b) in the Supporting Information]. The branching ratios of the four reaction channels are listed in Table 1. We also observe signatures of proton- and hydrogen-transfer channels (see Figure S1 in the Supporting Information), but these pathways cannot be resolved further.

**Figure 1:**
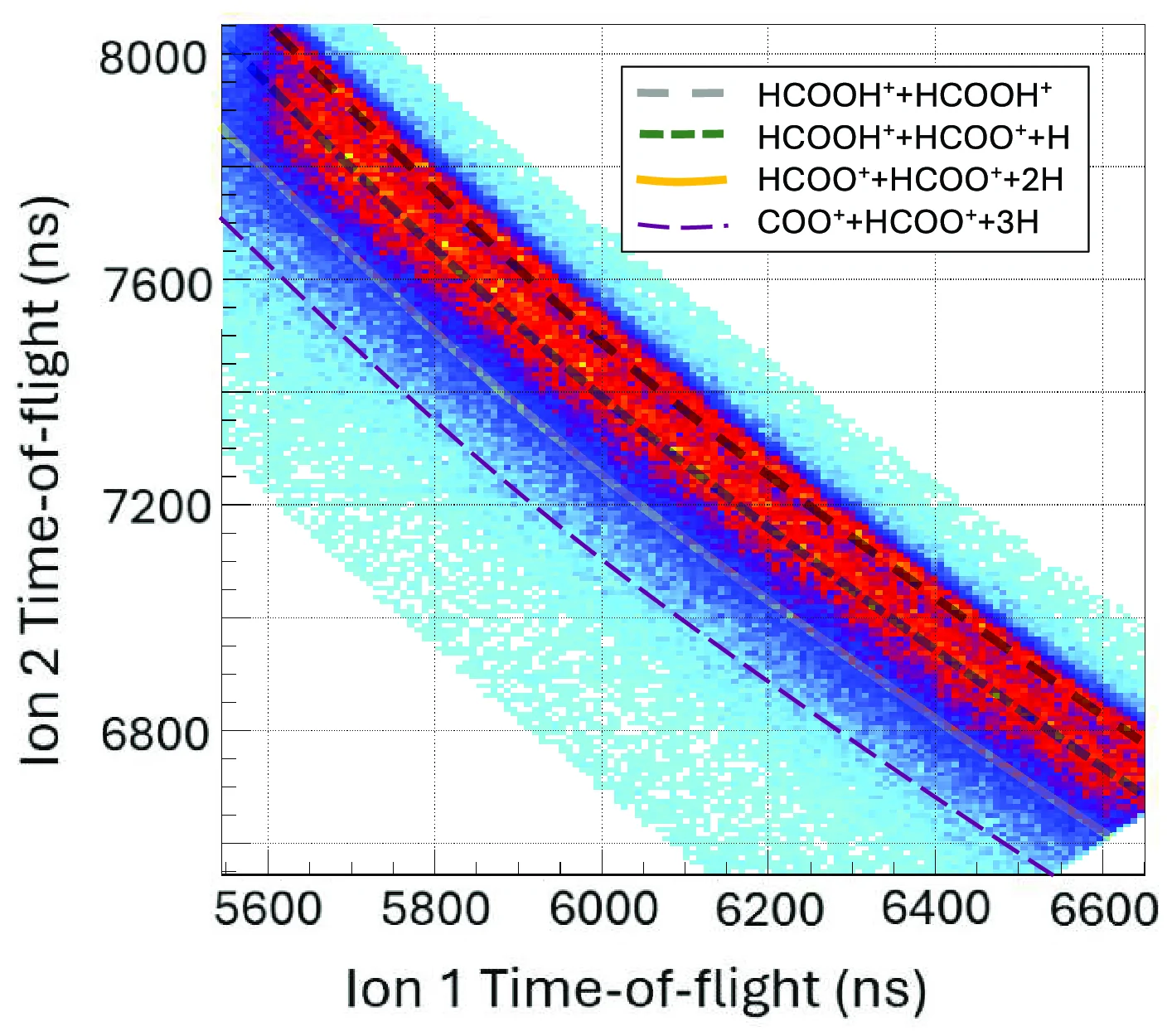
Zoomed in PIPICO spectrum of (HCOOH)_2_ with four fragmentation channels marked following photon absorption resulting in double ionization and subsequent dissociation -2-body breakup (gray dashed line, HCOOH^+^ + HCOOH^+^), 3-body breakup (green dotted line, HCOOH^+^ + HCOO^+^ + H), 4-body breakup (orange solid line, COOH^+^ + HCOO^+^ + 2H), and 5-body breakup (purple dashed line, CO_2_^+^ + HCOO^+^ + 3H). The channels are identified through peak fits in the sum TOF as detailed in the Supporting Information. The heatmap color scale is linear.

**Table 1:** Relative Yields of the 2-Body, 3-Body, 4-Body, and 5-Body Breakups of (HCOOH)_2_ upon Absorption of the 56 eV Photon Leading to the Double Ionization and Subsequent Dissociation Extracted from Fits to the TOF Sum of the Ion Pairs. See Figure S2­(b) in the Supporting Information ^a^

	Relative Yields (%)		
Channel	Total	DDI	SI
HCOOH^+^ + HCOOH^+^	25.3 ± 0.5	16 ± 3	9 ± 2
HCOO^+^ + HCOOH^+^ + H	41.9 ± 0.4	27 ± 5	15 ± 3
HCOO^+^ + HCOO^+^ + 2H	24.3 ± 0.9	–	–
CO_2_^+^ + HCOO^+^ + 3H	8.4 ± 2.8	–	–

a For the kinematically complete symmetric 2-body and asymmetric 3-body breakup channels, we also report the relative yields of DDI and SI. The experimental errors represent one standard deviation, accounting for only the statistical uncertainty. The separations of the breakup channels, their cross-contaminations, and identifications of DDI and SI contributions are described in detail in the Supporting Information.

We isolate the HCOOH^+^ + HCOOH^+^ and HCOOH^+^ + HCOO^+^ + H channels with narrow gates of ±50 ns around the orange dashed and green dotted lines in Figure 1; yet, cross contamination from neighboring reactions of up to 22% cannot be avoided [also see Figures S2­(a) and S2­(b) in the Supporting Information]. For the latter reaction channel, we can recover the momentum of the neutral H atom from the recoil of the center-of-mass of the detected ionic fragments (which is not possible for the 4- and 5-body breakups). The KERs of the symmetric fragmentation channel in eq 1 and asymmetric breakup in eq 2, on which we focus going forward, are calculated from all of their respective ionic and nuclear fragments.

The (HCOOH)_2_^2+^ yields for the symmetric 2-body and asymmetric 3-body channels are presented in Figure 2(a) and 2(b), as functions of the ion KERs and the electron eKEs. In both channels, the measured KERs peak near ∼4.15 eV, consistent with prior electron-impact ionization studies of (HCOOH)_2_.^25,26^ Compared to the symmetric breakup, the KER distribution for the asymmetric hydrogen elimination channel [see Figure 2(b)] is broader and more asymmetric, which is a result of fragmentation dynamics that involves breaking a valence bond and the release of a neutral hydrogen atom (also see Figure S3­(c) and S3­(d) in the Supporting Information). The observed lower correlated eKE is likely due to the higher total electronic binding energies, and the higher potential energies of the cation and dication states dissociating via the more complex three-body breakup process. This aligns with recent NA-AIMD simulations of the EUV double ionization in the presence of a strong NIR field that show that the asymmetric channel arises from excited dication states resulting in a lower electron energy sum (*E*_*e*_sum__) and in turn higher KER contributions.^28^ We see in Figure 2(c) that the *E*_*e*_sum__ contributions for the asymmetric channel are shifted by ∼1 eV to lower values.

**Figure 2:**
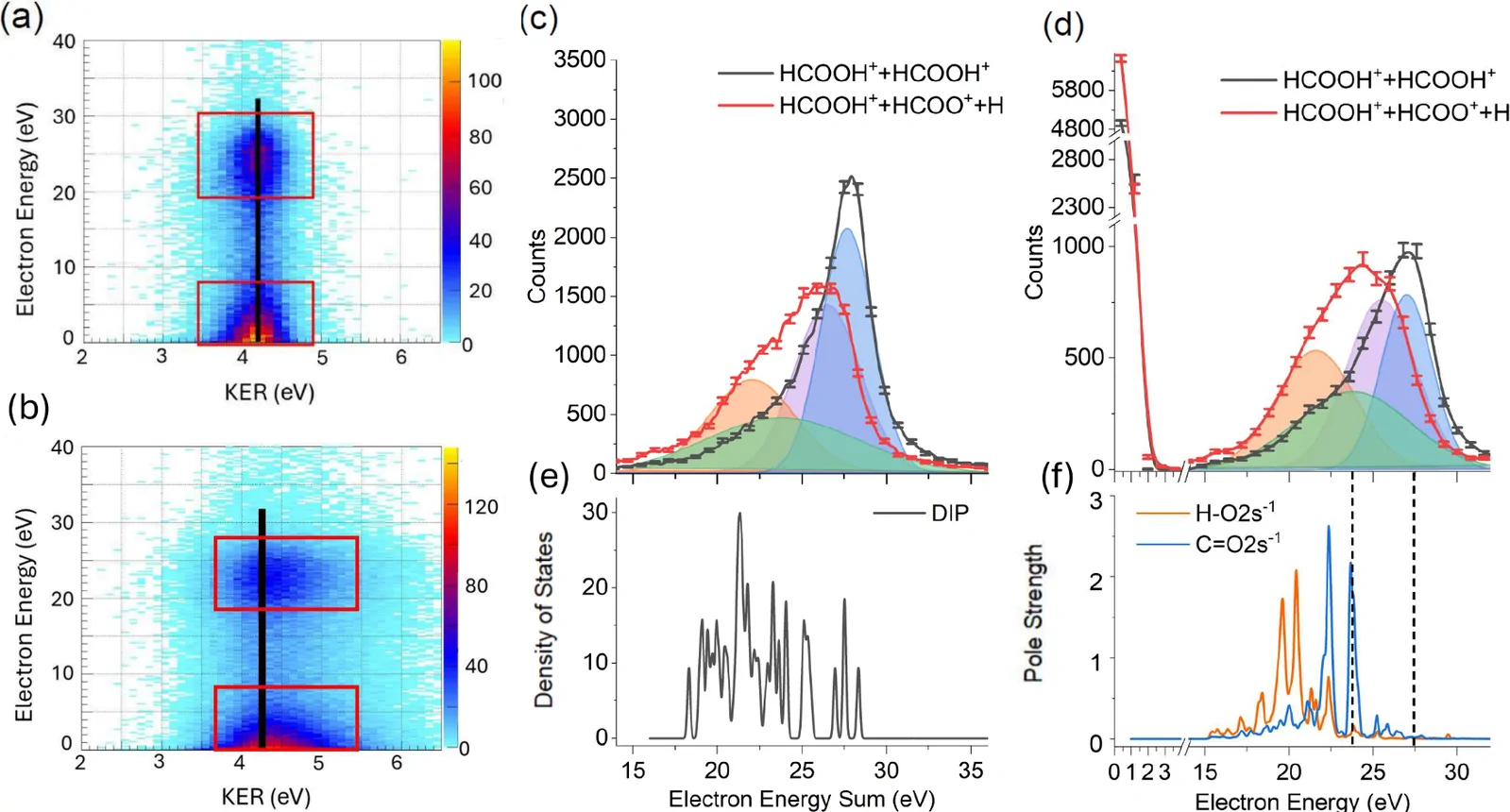
Measured KER and eKe shown as 2D electron-ion energy correlation heatmaps and 1D distributions. (a) Yield as a function of KER and eKE for the symmetric breakup channel and (b) for the asymmetric breakup channel. Red rectangles highlight LEEs and correlated photoelectrons from SI; black lines denote the continuous electron energy sharing of the DDI contribution. (c) The measured electron energy sum for the two channels. The fitted contributions to each curve from DDI and SI are depicted by the colored shaded regions (sym.-DDI, blue; sym.-SI, green; asym.-DDI, purple; asym.-SI, orange). (d) The measured high- and low-energy electron spectra for both channels. The fitted contribution to each curve from DDI and SI are depicted using the same color scheme as in panel (c). (e) The computed density of states of the energy difference between the photon and the dicationic states of (HCOOH)_2_. (f) The computed pole strengths of the energy difference between the photon and the cationic O­(2*s* ^–1^) states. [Carbonyl O­(2*s* ^–1^), blue; Hydroxyl O­(2*s* ^–1^), orange]. The vertical dashed lines at 23.35 and 27.56 eV in panel (f) correspond to the energy difference between the photon and the ground dicationic states of HCOOH and (HCOOH)_2_, respectively. The interval between them represents the pure ICD window. The heatmap color scale is linear. All error bars in panels (c) and (d) reflect one standard deviation of the statistical uncertainty.

A more-detailed comparison of the high- and low-energy electron energy distributions is provided in Figure 2(d), where it is evident that the electron kinetic energy (eKE) is accompanied by a spectral shift of the photoelectron peak from 26 eV for the symmetric channel to 23 eV for the asymmetric channel. This corresponds to the relevant photoelectron binding energies shifting from 30 eV in the symmetric fragmentation channel to 33 eV in the asymmetric channel.

As stated above, both DDI and SI processes result in the Coulomb explosion of the dimer dication, producing two charged fragments, as shown in the PIPICO map (see Figure 1). However, DDI and SI can be distinguished based on their electron energy sharing and electron angular distributions. In DDI, the two indistinguishable electrons share their kinetic energy uniformly, indicated by the vertical stripe spanning 0 to 30 eV in Figure 2(a) and 0 to 26 eV in Figure 2(b). Conversely, the SI events yield asymmetric electron energy distributions, with two distinct “heat islands” within each electron–ion energy correlation map. Here, the higher-energy photoelectron (*e*_*P*_), centered around 25 and 23 eV in the symmetric and asymmetric channel, respectively, is clearly separated from the LEE, which typically lies below 5 eV. These distinctions are further quantified by the electron energy sharing,

3 $$eE_{s}=\frac{E_{e_{1}}}{E_{e_{1}}+E_{e_{2}}}$$

illustrated in Figure S4 in the Supporting Information. For the symmetric and asymmetric fragmentation channels, the DDI to SI yield ratios are determined to be ∼9:5 for both channels, as shown in Table 1.

To separate the DDI and SI contributions, we analyzed both the electron energy sum [in Figure 2(c)] and the single-electron energy distributions [see Figure 2(d)]. The peak positions for DDI and SI were first determined from the two-dimensional electron energy correlation maps (see Figure S5 in the Supporting Information), and then refined through the normal distribution fits (see Figure S4 in the Supporting Information). Using the relative yields listed in Table 1 as constraints, the overall distributions were fitted with two components corresponding to the DDI and SI contributions. From these fits, the energy shift between the DDI and SI electron peaks was determined. As seen in Figure 2(c) and (d), the DDI component exhibits an electron energy sum peak at 27.6 eV and a photoelectron energy peak at 27.0 eV (blue shaded regions), whereas the SI component shows an electron energy sum peak at 24.5 eV and a photoelectron energy peak at 23.5 eV (green shaded regions). For the asymmetric channel, the DDI component peaks at 26.5 eV in the electron energy sum and 25.5 eV in the photoelectron spectrum (purple shaded regions), while the SI component peaks at 22.0 and 21.6 eV, respectively (orange shaded regions). Overall, an electron energy shift of approximately 3 to 4 eV is observed between the DDI and SI processes.

As a point of comparison, Figure 2(e) presents the calculated density of states of each electron energy sum obtained by subtracting the EOM-DIP-CCSD/cc-pVDZ (DIP) from the photon energy (56 eV). These values can be directly compared to the electron energy sum for DDI in Figure 2(c), but not in the case of SI. The computational results predict that DDI should become energetically viable for electron energy sums of 27.56 eV and above, which is in reasonable agreement with the measured onset of the DDI distributions and with previous work.^25^ The small deviation of the measured DDI distributions to higher electron energy sums is attributed to residual AI contamination and to the experimental particle energy resolution, which is approximately 0.1 × eKE. The shift of the distribution toward lower electron energy sums for the asymmetric channel compared to the symmetric channel indicates that excited doubly ionized states of (HCOOH)_2_ are more likely to contribute to the asymmetric channel following DDI.

SI can result from multiple electronic relaxation pathways, including AI and ICD, leading to the emission of low-energy secondary electrons. To obtain some insight into the energetic feasibility of AI versus ICD, we calculate the energies of the ground doubly ionized states of (HCOOH)_2_ and the formic acid monomer (HCOOH) at the EOM-DIP-CCSD/cc-pvdz level of theory. The lowest doubly ionized (HCOOH)_2_^2+^ state lies at 28.44 eV and corresponds to an electronic configuration with a positive charge on each monomer within the dimer. The doubly ionized state of the formic acid monomer lies higher in energy, at approximately 32.65 eV, but the adiabatic threshold of the dimer is close to the monomer value, within ±0.5 eV.^26^ Therefore, single ionized states of (HCOOH)_2_ that are above 28.44 eV have sufficient energy to directly decay through ICD into the charge-separated dicationic configuration of (HCOOH)_2_ → HCOOH^+^ + HCOOH^+^. The single ionized states with energy above 32.65 eV can additionally decay through intramolecular AI to the charge-localized dicationic configuration in which both charges lie on a single monomer (HCOOH + HCOOH^2+^). These configurations can contribute to the symmetric or asymmetric fragmentation channels through subsequent proton-transfer, hydrogen atom loss, or direct electron transfer.^26^

Figure 2(f) shows the calculated pole strengths for each eKE, obtained by subtracting the ADC(3)/cc-pVDZ single ionization spectrum from the photon energy. The spectrum, which correlates to the SI events but includes all possible fragmentation channels including those not producing two electrons, is decomposed into contributions arising from different functional groups. The higher photoelectron eKE peaks (lower binding energy) correspond to states with O­(2*s* ^–1^) character localized on the carbonyl oxygen (blue line), while the lower eKE peaks (higher binding energy) correspond to states with O­(2*s* ^–1^) character on the hydroxyl oxygen (orange line). Additionally, Figure 2(f) displays two dashed vertical lines at 27.56 and 23.35 eV, which correspond to the difference between the energy of the dicationic states of the formic acid dimer or monomer and the photon energy, respectively. Consequently, the regime *between* 23.35 and 27.56 eV corresponds to states that can *only* decay through ICD, while the regime *below* 23.35 eV correspond to states that can decay through *both* ICD or AI. Intriguingly, the pure ICD regime is most closely associated with states of carbonyl O­(2 s^–1^) character, while states of hydroxyl O­(2 s^–1^) character have sufficient energy to decay through both ICD or AI. The experimental SI contribution for both the symmetric and asymmetric channels in Figure 2(d) exhibit broad distributions that encompass ionization of both oxygen sites. Note that our aforementioned electron energy resolution of Δ*E* ≈ *E*/10 prevents resolving any of the sharp peaks of Figure 2(f) . However, the shift of the asymmetric channel toward lower eKE indicates a greater contribution to this channel from hydroxyl oxygen ionization and therefore, a mixture of ICD/AI. In comparison, the peak-maximum of the symmetric channel is well-aligned with ionization of the carbonyl oxygen and therefore predominantly decays through pure ICD. A similar energy balance analysis was performed previously,^25^ but here we emphasize the energy difference between the two types of O­(2 s^–1^) states and make a more direct comparison to the symmetric and asymmetric channels.

To corroborate this computational analysis and experimentally determine whether AI is an energetically accessible and active pathway in (HCOOH)_2_^2+^, several 2-body fragmentation channels of the HCOOH monomer were briefly examined. At 56 eV, multiple dicationic states are accessible in the HCOOH monomer, leading to fragmentation channels such as HCOO^+^ + H^+^ and H_2_O^+^ + CO^+^ [see eqs (S1) and (S5), respectively, in the Supporting Information]. For a full overview, see Figure S1 in the Supporting Information The analysis of the electron energy sharing for those channels suggests contributions from both DDI and SI in the monomer fragmentation, which is consistent with possible AI pathways (see Figures S6 and S7 in the Supporting Information). These findings are in agreement with earlier electron-impact ionization studies for formic acid monomers.^41^ Consequently, intramolecular AI represents a plausible decay pathway in (HCOOH)_2_^+^ alongside ICD.

To further support our findings, we also investigated the measured electron and ion angular distributions, which are presented in the Supporting Information (see Figures S8, S9, and S10). In brief, the isolated photoelectrons exhibit a moderate anisotropy along the light polarization axis, whereas the low-energy secondary electrons are nearly isotropic, consistent with sequential AI/ICD decay processes. While the symmetric breakup shows no preferred fragmentation direction with respect to the light polarization, the asymmetric channel displays an anisotropy for DDI that suggests prompt bond-directed dissociation, whereas SI remains largely isotropic, indicative of a slower, stepwise fragmentation. For the SI, we report the anisotropy parameters for the photoelectrons and LEEs for the symmetric and asymmetric fragmentation channels as well as their relative yields in Table 2, using 23 eV as the cutoff energy between the pure ICD regime (ICD-Only) and the regime in which both ICD and AI are energetically feasible (AI/ICD).

**Table 2:** Fitted Asymmetry Parameters β of the Photoelectron *e*_*P*_ and the Sequentially Emitted Second Electron *e*_LEE_ for the Separated Mixed AI/ICD and ICD-Only Contributions to the SI Ionization Mechanisms for Symmetrical and Asymmetrical (HCOOH)_2_ Breakup upon Absorption of a 56 eV EUV Photon

Channel	Mechanism	β_*e*_*p*__	β_*e*_LEE__	Relative Yield (%)
Sym.	AI/ICD	0.728 ± 0.0340	0.0312 ± 0.0207	9.63 ± 0.87
Sym.	ICD-Only	0.614 ± 0.0126	–0.0316 ± 0.00981	33.88 ± 1.45
Asym.	AI/ICD	0.676 ± 0.0138	0.0137 ± 0.0149	23.79 ± 1.39
Asym.	ICD-Only	0.671 ± 0.0142	0.0565 ± 0.0111	32.68 ± 1.59

To examine the time-scale and competition between the relaxation processes during the SI mechanism, Figure 3 presents explicit real-time simulations of the electron dynamics in (HCOOH)_2_ using our RT-TDDFT methodology.^32^ In this initial investigation, the nuclei are held fixed in space, as the final Coulomb explosion occurs on a longer time-scale.^25,28^ However, coupled electron–nuclear processes can have an influence on electron relaxation, as observed, for example, in the water dimer.^7–10,42,43^ We analyze initial ionization of either the carbonyl or hydroxyl O­(2s) molecular orbital (MO), which correspond to the localized **5a′** or **4a′** MO on one of the two monomers, respectively. We label this initially ionized monomer as monomer-A and the other as monomer-B. These calculations reveal the ultrafast (<20 fs) nature of the process and help bridge the gap between the static energetics of the cationic vs dicationic states and the longer time-scale dynamics of the Coulomb explosion event explored in previous work.^25^

**Figure 3:**
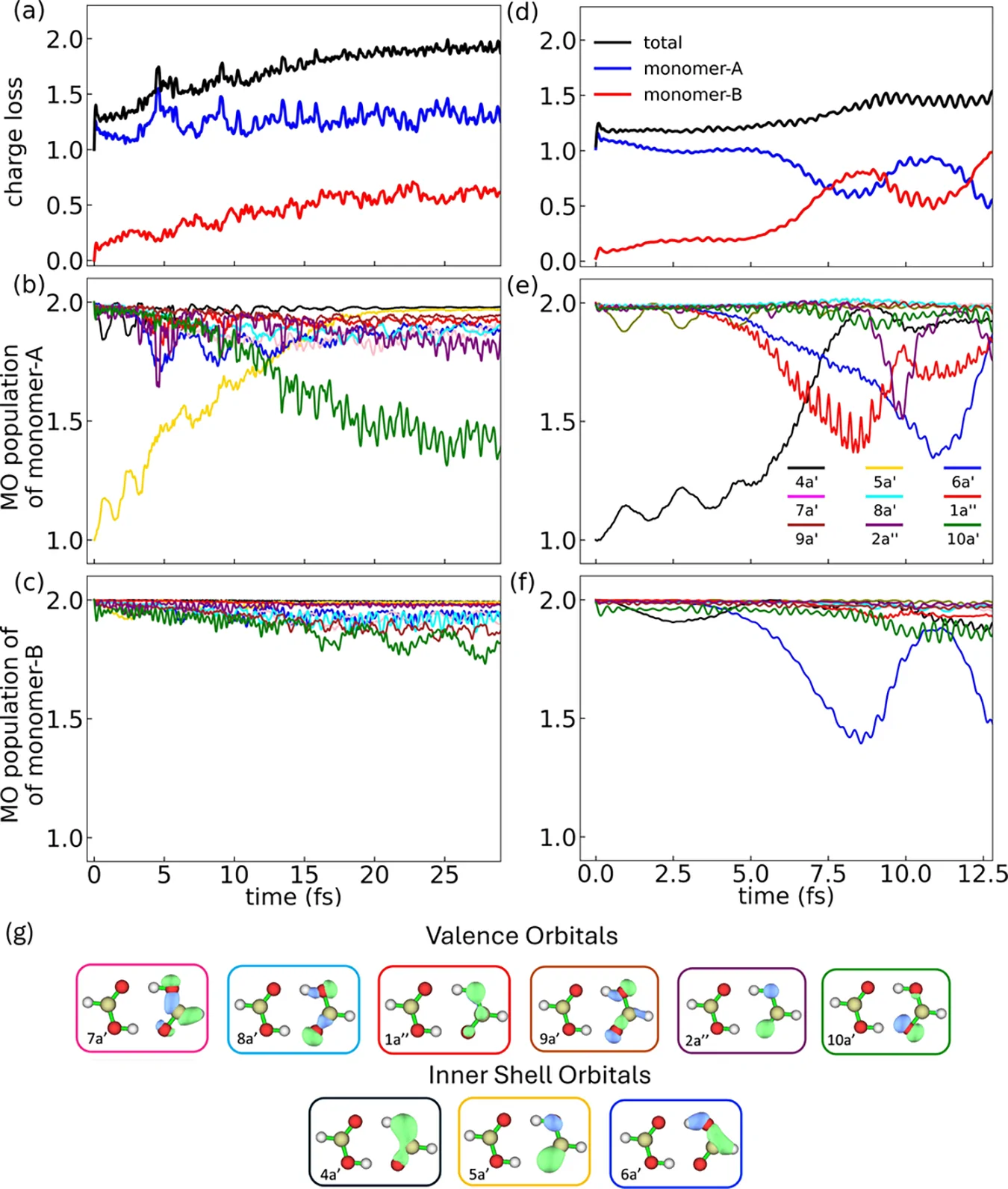
RT-TDDFT results on (HCOOH)_2_ following ionization of either the (a)–(c) **5a′** or (d)–(f) **4a′** MO of monomer-A. (a) and (d) The time-dependent charge-loss on the two monomers (monomer-A in blue and monomer-B in red) in (HCOOH)_2_ and the total charge loss of (HCOOH)_2_ (black). The time-dependent population of the MOs in the localized monomer basis associated with monomer-A are plotted in (b) and (e) and those associated with monomer-B in (c) and (f). The same color scheme is used in all four plots as labeled in panel (e). (g) Images of the corresponding MOs on monomer-A plotted using Multiwfn.^44^ The symmetrically equivalent MOs on monomer-B are found in Figure S11 of the Supporting Information.

Figure 3(a) shows that the charge loss on monomer-A following **5a′** ionization starts at one and remains relatively steady throughout the simulation. In comparison, the charge loss on monomer-B starts at zero and steadily increases on the same time scale as the total (HCOOH)_2_ system loses charge, as expected for an ICD mechanism. This is in agreement with the energy picture presented in Figure 2(f), in which the main carbonyl O­(2 s^–1^) peaks are either in the ICD-only or only slightly into the mixed AI/ICD regimes. The total charge loss in Figure 3(a) plateaus around 20 fs. The final charge distribution of the two monomers contains significant positive charges on both monomers, consistent with a Coulomb explosion producing two singly charged HCOOH^+^ monomers. We should emphasize that the simulations are performed with fixed nuclei, as the focus of the theoretical calculations is on the electron dynamics and not on distinguishing between the two fragmentation channels.

To further confirm that the relaxation dynamics following ionization of the **5a′** MO correspond to ICD, Figures 3(b) and (c) depict the populations of the localized MOs on monomers A and B, respectively. Figure 3(b) shows that an initial hole is generated in the **5a′** MO of monomer-A. This orbital is repopulated by electron density relaxing primarily from the monomer-A **10a′** MO, an oxygen 2p-like valence orbital. Figure 3(c) shows a simultaneous loss of electron density from most valence orbitals on monomer-B. The summed population loss across these orbitals matches the charge depletion on monomer-B observed in Figure 3(a). This behavior is somewhat distinct from previous work, in which secondary ionization was dominated by one or two valence orbitals.^32^ However, the more complex electronic structure of (HCOOH)_2_, with its closely spaced valence MOs, is the most likely contributor to this behavior. Nevertheless, the analysis does support ICD.

Analogous electron dynamics following ionization of the **4a′** MO are presented in Figures 3(d–f). As expected, they exhibit more complex behavior because the hydroxyl O­(2 s^–1^) peaks in Figure 2(f) lie firmly within the mixed AI/ICD regime. Specifically, Figure 3(d) shows that secondary ionization of (HCOOH)_2_ occurs on a faster time-scale; the total charge loss begins to increase at 7 fs and plateaus after 9 fs. The plateau value is below that in Figure 3(a) and the expected value of 2.0, as has been observed in our previous work.^32^ A perfect final value of 2.0 is not expected due to the approximate nature of the RT-TDDFT methodology, especially for the more complicated relaxation dynamics following ionization of the **4a′** MO. Additionally, the secondary ionization occurs simultaneously with resonant charge transfer observed by the out-of-phase oscillations of the charge loss.

The time-dependent MO populations show that the initial hole in the **4a′** MO of monomer-A [Figure 3(e)] is repopulated on a similar time-scale to loss of electron density from the **1a′′** and **6a′** MOs on monomer-A and the **6a′** MO on monomer-B [Figure 3(f)]. Interestingly, part of this dynamics occurs before secondary ionization begins at 7 fs and is therefore associated with the start of the resonant charge transfer. In the 7–9 fs range, when secondary ionization is observed to occur, there is a rapid increase in the charge loss on monomer-B that is not fully compensated by a decrease in the charge loss of monomer-A [Figure 3(f)]. This indicates that the continuous decrease of the **6a′** MO population on monomer-B, in this time-window, is associated with ionization of this MO, which is indicative of an ICD process. However, there is additionally a population decrease of the **10a′** and **2a′′** MO on monomer-A [Figure 3(f)] only within this time window, which indicates that there is some contribution of AI of monomer-A as well. Following secondary ionization, (HCOOH)_2_ continues to exhibit resonant charge transfer between the **6a′** MOs on each of the two monomers. The oscillations are expected to decay in the presence of nuclear dynamics, as the monomers break apart.

Lastly, Figure 4 shows the time-dependent real-space hole density following ionization of the **4a′** MO. Both, the charge and energy transfer between the two monomers during the electron relaxation dynamics occur across the diagonally adjacent oxygen atoms bonded directly to the hydrogen atoms instead of along the hydrogen bonds. The charge transfer can be attributed to the degeneracy of the corresponding orbitals. However, somewhat surprisingly, the energy transfer also occurs along the diagonal, even though ICD does not occur through degenerate orbitals. This could help explain the quick rotation of the two hydrogen atoms involved in the hydrogen bonding observed in previous *ab initio* molecular dynamics simulations and hints at possible future chemical modifications to affect the behavior of electron relaxation within (HCOOH)_2_.^25^ This in-depth investigation of the ultrafast dynamics illustrates the uniqueness of the RT-TDDFT methodology to unveil fine details of the electron relaxation dynamics.

**Figure 4:**
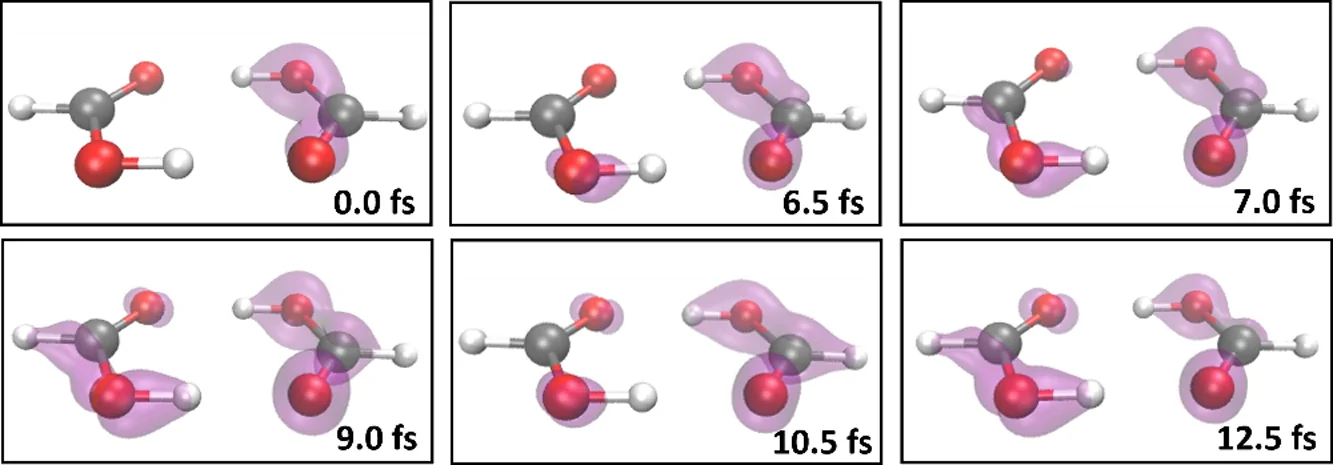
Snapshots of the time-dependent real-space hole density plotted as a three-dimensional isosurface superimposed on the nuclear geometries of monomer-A on the right and monomer-B on the left following ionization of the **4a′** MO of monomer-A. The frames depict different times along the RT-TDDFT trajectory.

In summary, we have resolved the ultrafast dissociation dynamics of the formic acid dimer following 56 eV EUV ionization. Focusing on the symmetric 2-body and asymmetric 3-body breakup channels, we quantified the relative contributions of DDI and SI mechanisms. In both reaction channels, DDI dominated, in agreement with recent EUV/strong-field-NIR pump–probe experiments.^28^ Our results demonstrate that SI is driven by a competition between AI and ICD, where the decay pathway is highly sensitive to the initial vacancy location. RT-TDDFT simulations support this interplay, placing the electronic relaxation within a 20 fs window. Intriguingly, the simulations also indicate that charge and energy transfer occurs across the diagonally adjacent oxygen atoms instead of along the hydrogen bonds.

These findings establish local AI and nonlocal ICD as primary decay mechanisms for SI in weakly bound hydrogen-bonded systems, offering a holistic view of electron-driven fragmentation relevant to DNA base-pair interactions. While this work focused on electronic relaxation in SI at fixed nuclear geometries, future studies employing time-resolved pump–probe spectroscopy and nonadiabatic simulations will be essential to track the time-evolution of these processes in single unperturbed molecular dimers. Recent EUV/strong-field-NIR investigations of formic acid dimers suggest that the relevant time scales are as long as 250 fs,^27,28^ and our investigations indicate processes as short as 20 fs. Such ultrafast particle coincidence investigations will clarify the role of nuclear motion, such as proton transfer, in mediating charge redistribution in complex biological environments.^45–52^

## Methods

### Experiment

The experimental data were obtained at the open port of beamline 10.0.1 of the Advanced Light Source (ALS), located at Lawrence Berkeley National Laboratory (LBNL). A reaction microscope, i.e., a Cold Target Recoil-Ion Momentum Spectroscopy (COLTRIMS) roll-up end-station was used to obtain 3-D electron and ion momenta in coincidence on an event-by-event basis. In the experiment, the 3-D momenta of detected ions and electrons were calculated using the measured TOF and the position of impact of the particles. The monomer ionization and fragmentation data were collected simultaneously with the dimer ionization data. True coincident events of the dimer fragmentation of interest in this analysis contributed only on the order of 1% to 2%, relative to the monomers.^30,31^ The data are analyzed and visualized by ROOT and LMF2ROOT.^53^ Further details of the experimental setup like the spectrometer geometry, electric and magnetic guiding fields, as well as the target preparation, can be found in the Supporting Information.

### Computation

The RT-TDDFT calculations were initialized from ground-state density functional theory (DFT) calculations on (HCOOH)_2_ in its lowest-energy double hydrogen-bonded geometry previously obtained at the CCSD­(T)/aug-cc-pVTZ level of theory.^54^ All calculations were performed using the range-separated LC-PBE*functional using a tuned range-separated parameter and the aug-cc-pVDZ basis set. The explicit real-time dynamics of (HCOOH)_2_ are simulated using RT-TDDFT including a complex absorbing potential (CAP) to effectively remove the secondary ionized electron from the system based on the following equations of motion for the 1-electron reduced density matrix (1RDM)^32,55,56^

4 $$i\frac{\partial P^{\prime }\left(t\right)}{\partial t}=F^{\prime }\left(t\right)P^{\prime }\left(t\right)-P^{\prime }\left(t\right){F^{\prime }}^{†}\left(t\right)$$

Here, **P**′(*t*) is the 1RDM and **F**′(*t*) is the modified non-Hermitian DFT Fock matrix,

5 $$F^{\prime }\left(t\right)=F_{0}^{\prime }\left(t\right)+i\Gamma^{\prime }\left(t\right)$$

The prime notation indicates that the matrices were defined in the basis of orthogonalized atomic orbitals (AOs).^57^ The CAP was implemented through the inclusion of the damping matrix, **Γ** ^′^(*t*). A time step of 1 au was used for the RT-TDDFT calculations. Our time-dependent electronic structure (TiDES) code was used to perform all calculations.^58^ Full details and the values of all necessary parameters follow from previous work and are defined in the Supporting Information.^32^

## Supplementary Material



## Notes

Information is provided on the analysis of the experimental data, including the identification and isolation of reaction channels for the target monomer and dimer, determination of branching ratios, visualization of the energetics, separation of SI from DDI and AI from ICD, possible SI and DDI dissociation pathways, and electron and ion angular distributions. Additional details on the experimental setup are also provided. Furthermore, the Supporting Information elaborates on the choice of range-separation and CAP parameters and the definition of observables used in the RT-TDDFT calculations (PDF)
