# Supplementary material for: Dynamics and Energy Relaxation of Formic Acid Dimers Following Inner Valence Ionization
Source: J Phys Chem Lett. 2026 Oct 5;17(41):11896–905. doi: 10.1021/acs.jpclett.6c03064 (PMC13679088; doi:10.1021/acs.jpclett.6c03064)
Supplement: Supplementary file 1 [file jz6c03064_si_001.pdf]

Supporting Information: Dynamics and Energy Relaxation of Formic Acid Dimers Following Inner Valence Ionization

J.X. Zhong Manis,[†] Y.S. Wang,[†,‡] M.C. Rohan,[†] S. Kumar,[¶] M. Shaikh,[¶] J. Stindl,[§] G. Kastirke,[§] O.D. McGinnis,[§] M. Kircher,[§] C. Bagdia,[‖] N. Iwamoto,[‖] R. Enoki,[⊥] V. Davis,[⊥] J.B. Williams,[⊥] R. Dörner,[§] I. Ben-Itzhak,[‖] D.S. Slaughter,[¶] T. Weber,[*,¶] J.S. Kretchmer,[*,†] and T.M. Orlando[*,†,#]

†*School of Chemistry and Biochemistry, Georgia Institute of Technology, Atlanta, GA, 30332, USA*

‡*Tamkang University, Department of Chemistry, Tamsui, New Taipei City, 251301, Taiwan*

¶*Chemical Sciences Division, Lawrence Berkeley National Laboratory, Berkeley, CA, 94720, USA*

§*Institut für Kernphysik, Goethe-Universität, 60438, Frankfurt am Main, Germany*

‖*J. R. Macdonald Laboratory, Department of Physics, Kansas State University, Manhattan, KS, 66506, USA*

⊥*Department of Physics, University of Nevada, Reno, NV, 89557, USA*

#*School of Physics, Georgia Institute of Technology, Atlanta, GA, 30332, USA*

E-mail: TWeber@lbl.gov; jkretchmer@gatech.edu; thomas.orlando@chemistry.gatech.edu

This section contains supporting information on the analysis of the experimental data to identify and isolate the reaction channels of the target monomer and dimer, determine

the branching ratios, visualize the energetics, separate SI from DDI and AI from ICD, lists possible SI and DDI dissociation pathways, discusses the electron and ion angular distributions, and provides more details on the experimental setup. Moreover, we elaborate the details of the choice of range-separation and CAP parameters as well as the definition of the observables for the RT-TDDFT calculations.

1. Possible symmetric 2-body and asymmetric 3-body dimer fragmentation pathways after photo double ionization

We present selected pathways for the 2-body and 3-body dissociation of the formic acid dimer following direct double ionization (DDI) and sequential ionization (SI) in Scheme 1.

Direct Double Ionization (DDI)

$h\nu + (HCOOH)_2 \rightarrow 2e + (HCOOH)_2^{2+} \rightarrow HCOOH^+ + HCOOH^+$

$2e + (HCOOH)_2^{2+} \rightarrow HCOOH^+ + HCOO^+ + H$

$2e + (HCOOH)_2^{2+} \rightarrow HCOOH^+ + HCOOH^{+*} \rightarrow HCOOH^+ + HCOO^+ + H$

$2e + (HCOOH)_2^{2+} \rightarrow HCOOH + HCOOH^{2+} \xrightarrow{(PT)} H_2COOH^+ + HCOO^+ \rightarrow HCOOH^+ + H + HCOO^+$

Sequential Ionization (SI)

$h\nu + (HCOOH)_2 \rightarrow e_P + (HCOOH + HCOOH^{+*}) \rightarrow e_{ICD} + HCOOH^+ + HCOOH^+$

$e_P + (HCOOH + HCOOH^{+*}) \rightarrow e_{ICD} + HCOOH^+ + HCOO^+ + H$

$e_P + (HCOOH + HCOOH^{+*}) \xrightarrow{(HT)} HCOO^{+*} + H_2COOH \rightarrow e_{ICD} + HCOO^+ + H_2COOH^+ \rightarrow HCOO^+ + HCOOH^+ + H$

$e_P + (HCOOH + HCOOH^{+*}) \xrightarrow{(HT)} HCOO^{+*} + HCOOH + H \rightarrow e_{ICD} + HCOO^+ + HCOOH^+ + H$

$e_P + (HCOOH + HCOOH^{+*}) \xrightarrow{(PT)} HCOO^{*} + H_2COOH^+ \rightarrow e_A + HCOO^+ + HCOOH^+ + H$

$e_P + (HCOOH + HCOOH^{+*}) \xrightarrow{(PT)} HCOO^{*} + H_2COOH^+ \rightarrow e_A + HCOO^+ + H_2COOH^+ \xrightarrow{(HT)} HCOOH^+ + HCOOH^+$

Scheme 1: Breakup pathways of the formic acid dimer upon 56 eV photo-absorption initiating DDI (blue) or SI (red). Symmetric (green) and asymmetric (orange) fragmentation channels are shown, together with possible relaxation mechanisms for SI [inter-molecular Coulombic decay (ICD), autoionization (A), proton transfer (PT), and hydrogen transfer (HT)].

2.1 Experiment: Identification of the reaction channels of the formic acid target and separation of the dimer breakup channels

In Fig. S1 we show the PIPICO spectrum for the supersonic formic acid gas jet target, which contains about 3% dimers. We can identify the following reaction channels of the formic acid monomer (with "N" denoting the undetected neutral fragment or fragment combinations):

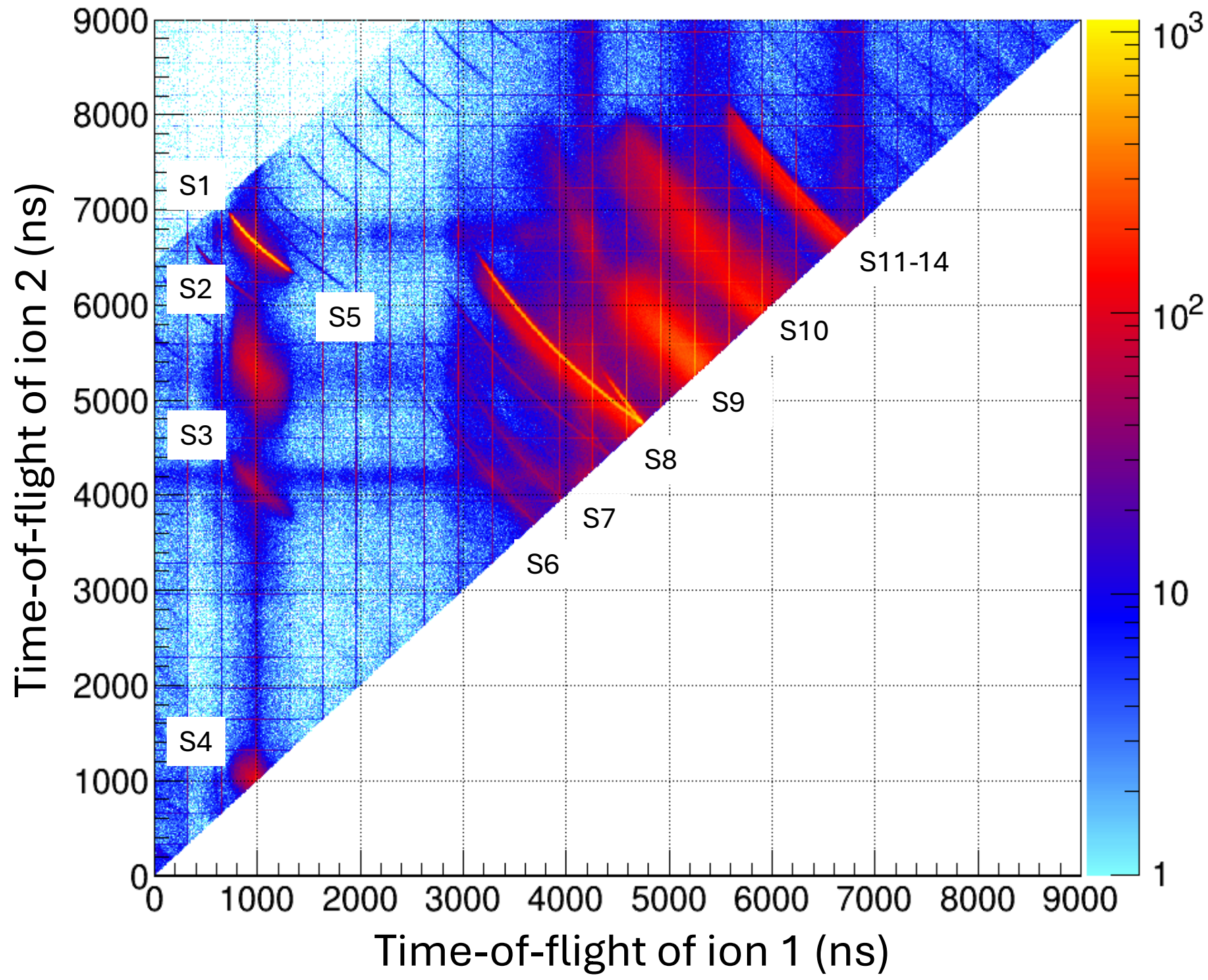

Figure S1: The full PIPICO spectrum in log-scale intensity of the collected monomer and dimer channels upon photo double ionization with 56 eV of the formic acid target with a coarse momentum conservation constraint applied. The identified channels are listed in the text as Eqs. (S1) to (S14). The heatmap color scale is logarithmic.

$$
\begin{aligned}
&HCOOH + h\nu(56\ eV) \\
&\rightarrow H^+ + HCOO^+ + 2e^- && \text{(S1)} \\
&\rightarrow H^+ + HCO^+ + O + 2e^- && \text{(S2)} \\
&\rightarrow H^+ + OH^+ + N + 2e^- && \text{(S3)} \\
&\rightarrow H^+ + H^+ + N + 2e^- && \text{(S4)} \\
&\rightarrow H_2^+ + CO_2^+ + 2e^- && \text{(S5)} \\
&\rightarrow N^+ + N^+ + 2e^- && \text{(S6)} \\
&\rightarrow O^+ + O^+ + 2e^- && \text{(S7)} \\
&\rightarrow O^+ + HCO^+ + H + 2e^- \\
&\rightarrow O^+ + HCOH^+ + 2e^- \\
&\rightarrow OH^+ + HCO^+ + 2e^- \\
&\rightarrow \underline{H_2O^+ + CO^+ + 2e^-} && \text{(S8)}
\end{aligned}
$$

The most prominent reaction channel in the brown family of Eq. (S8) is the underlined pathway $H_2O^+ + CO^+ + 2e^-$. Reaction channels Eqs. (S6) and (S7) are from the residual background, and the dissociation pathway Eq. (S4) is also polluted by double ionization events from the remaining water vapor in the vacuum chamber. For the dissociation of the dimers, we identify the following reaction channels:

$$
\begin{aligned}
&(HCOOH)_2 + h\nu\ (56\ eV) \\
&\rightarrow CO^+ + CO^+ + N + 2e^- \\
&\rightarrow CO^+ + HCO^+ + N + 2e^- \\
&\rightarrow HCO^+ + HCO^+ + N + 2e^- \\
&\rightarrow HCO^+ + H_2CO^+ + N + 2e^- \\
&\rightarrow H_2CO^+ + H_2CO^+ + N + 2e^- && \text{(S9)} \\
&\rightarrow (O_2^+ + CCO^+ + N + 2e^-) \\
&\rightarrow (H_2O_2^+ + CCO^+ + N + 2e^-) \\
&\rightarrow (H_3O_2^+ + CCO^+ + N + 2e^-) \\
&\rightarrow (H_2O_2^+ + HCCO^+ + N + 2e^-) \\
&\rightarrow (H_3O_2^+ + HCCO^+ + N + 2e^-) \\
&\rightarrow \underline{CHO^+ + HCOOH^+ + N + 2e^-} && \text{(S10)} \\
&\rightarrow CO_2^+ + HCOO^+ + 3H + 2e^- && \text{(S11)} \\
&\rightarrow HCOO^+ + HCOO^+ + 2H + 2e^- && \text{(S12)} \\
&\rightarrow HCOOH^+ + HCOO^+ + H + 2e^- && \text{(S13)} \\
&\rightarrow HCOOH^+ + HCOOH^+ + 2e^- && \text{(S14)}
\end{aligned}
$$

The most likely reaction channel in the red family of Eq. (S10) is the underlined pathway $CHO^+ + HCOOH^+ + N + 2e^-$, which has been observed in recent EUV/strong-field-NIR

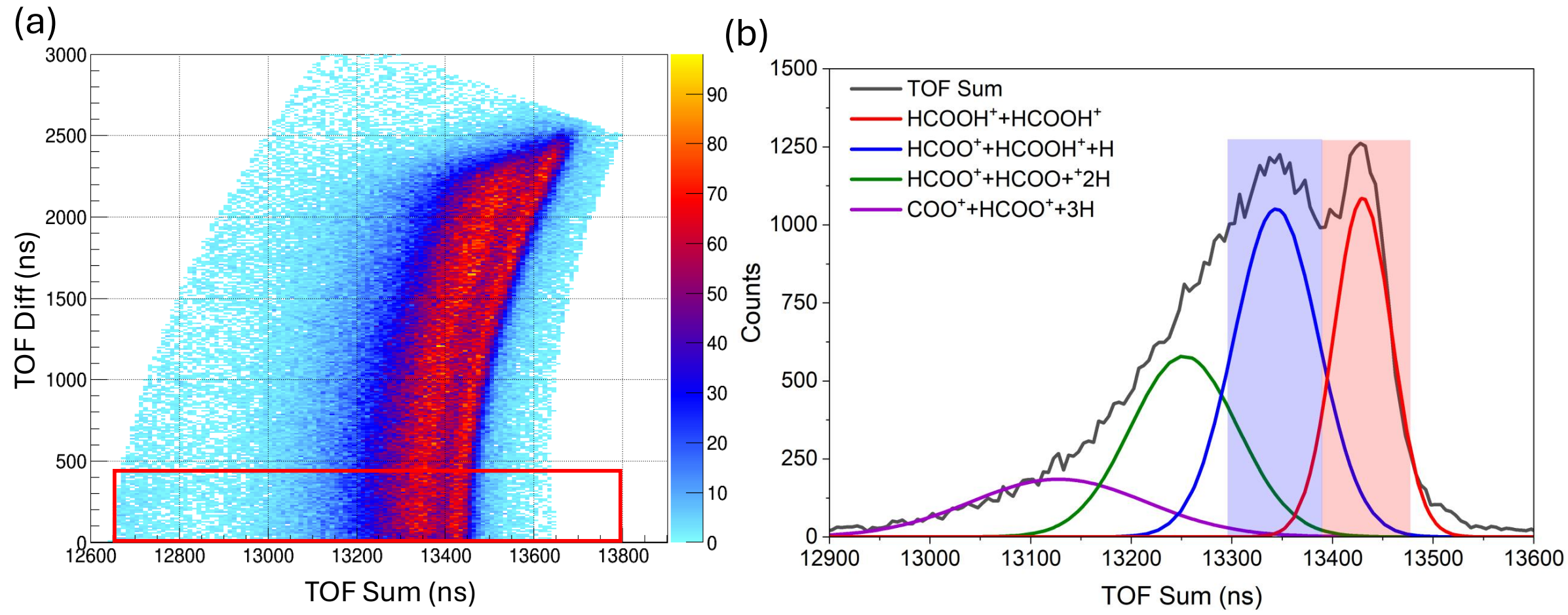

Figure S2: (a) Time-of-flight Difference as a function of the Time-of-flight Sum of the recorded dimer fragment-ion pairs. The heatmap color scale is linear. (b) Four-peak Gaussian fit of the ion-pair time-of-flight sum for a 0 - 400 ns gate in the TOF Difference in order to separate the individual contributions to the PIPICO spectrum. The red and blue shaded region indicate the software gates applied for 2- and 3-body analysis in the main text.

pump-probe studies.[1] This observation suggests that the dimer dication accesses excited states and undergoes complex 3-body fragmentation involving C–O bond cleavage, H migration, and maybe OH roaming. As mentioned in the main text, the same work also reports the $HCOOH^+ + H_2O^+ + CO$ channel, which we do not observe.[2] We also do not detect signatures of long lived dimer dications that dissociate on the microscale timescale, as recently reported.[1] This suggests that strong- and weak-field double ionization of the formic acid dimer may favor different fragmentation pathways. Consistent with this picture, we observe only negligible contributions from $CO_2^+ + CO_2^+ + N$, a channel previously suggested following irradiation with short (100 fs), 800 nm NIR pulses at $1 \cdot 10^{14}$ W/cm^2.[3] While not excluded, as they match the broad PIPICO island, the other channels in the red family of Eq. (S10) appear rather exotic, as they would require substantial intramolecular rearrangement to convert the two formic-acid monomers into a peroxide-like ionic fragment and a ketenyl cation before or during dication dissociation. We thus only list them here for completeness and, hence, in brackets. On the other hand, the PIPICO feature assigned to Eq. (S10) is remarkably broad. If the $CHO^+ + HCOOH^+ + N$ channel is its sole contributor, the undetected OH fragment, or alternatively O + H, must carry away a substantial fraction of the available kinetic energy. This is plausible, given the relatively low masses of these neutral fragments in this fragmentation scenario.

Fig. 1 in the main text shows the section of the PIPICO spectrum that identifies the detected dimer breakup channels listed in Eqs. (S11) to (S14). In Fig. S2(b), we show a projection of that PIPICO feature to the TOF sum of the two detected fragment ions for a small slice of the TOF difference of the ions pairs (0 to 400 ns), as shown in Fig. S2(a). A four-gaussian peak fit centered on the TOF sums that are correlated to the PIPICO lines in Fig. 1

of the main text reveal the ratios. We repeat this fitting procedure for six concatenated slices of the the TOF difference to cover the entire PIPICO feature and calculate the average to gain the branching ratios that are presented in Table. 1 in the main text. For the symmetric 2-body and asymmetric 3-body breakup pathways of interest in this study [red and blue curves in Fig. S2(b), respectively], we apply software gates of ±50 ns around the TOF sums to isolate those channels. The maximum cross-contamination from the asymmetric 3-body channel is estimated to be about 21.8% for the 2-body symmetrical breakup, and 21.5% from the 2-body and 4-body channels for the asymmetric 3-body breakup in the Fig. S2 using the peak fits and gates applied in the blue and red shaded regions.

2.2 Experiment: Electron-ion energy correlation maps, $E_{e_{sum}}$ and KER distributions of the symmetric 2-body and asymmetric 3-body dimer reaction channels

In Fig. S3(a) and (b), we show the electron–ion energy-correlation maps, with the electron energy sum ($E_{e_{sum}}$) plotted as a function of the kinetic energy release (KER) for the symmetric 2-body and asymmetric 3-body channels, respectively. The distribution in panel (a) is well localized, suggesting that only a limited number of dication states and dissociation limits contribute to the 2-body breakup. In contrast, the distribution in panel (b) is broader and shifted toward lower $E_{e_{sum}}$, indicating contributions from excited dication states. In Fig. S3(c) and (d), we show the corresponding KER distributions for the symmetric 2-body and asymmetric 3-body channels. The red curves represent the contributions from SI, while the blue curves show the contributions from DDI. The SI gates are indicated by the red rectangles in Fig. 2(a) and (b) of the main text, with the regions between these gates corresponding to the DDI contribution. The black curves show the KER distributions for all events contributing to the respective fragmentation channels. Overall, both the KER and $E_{e_{sum}}$ distributions are relatively sharp for the 2-body channel and substantially broader for the 3-body channel. We find little difference between the FWHM KER widths of the DDI and SI contributions. The corresponding $E_{e_{sum}}$ distributions are shown in Fig. 2(e) and (f) for the symmetric 2-body and asymmetric 3-body channels, respectively. These distributions reveal the broad SI (red) and narrower DDI (blue) contributions. The spectral shift between the two fragmentation channels provides further evidence for the population of excited dication states in the 3-body channel.

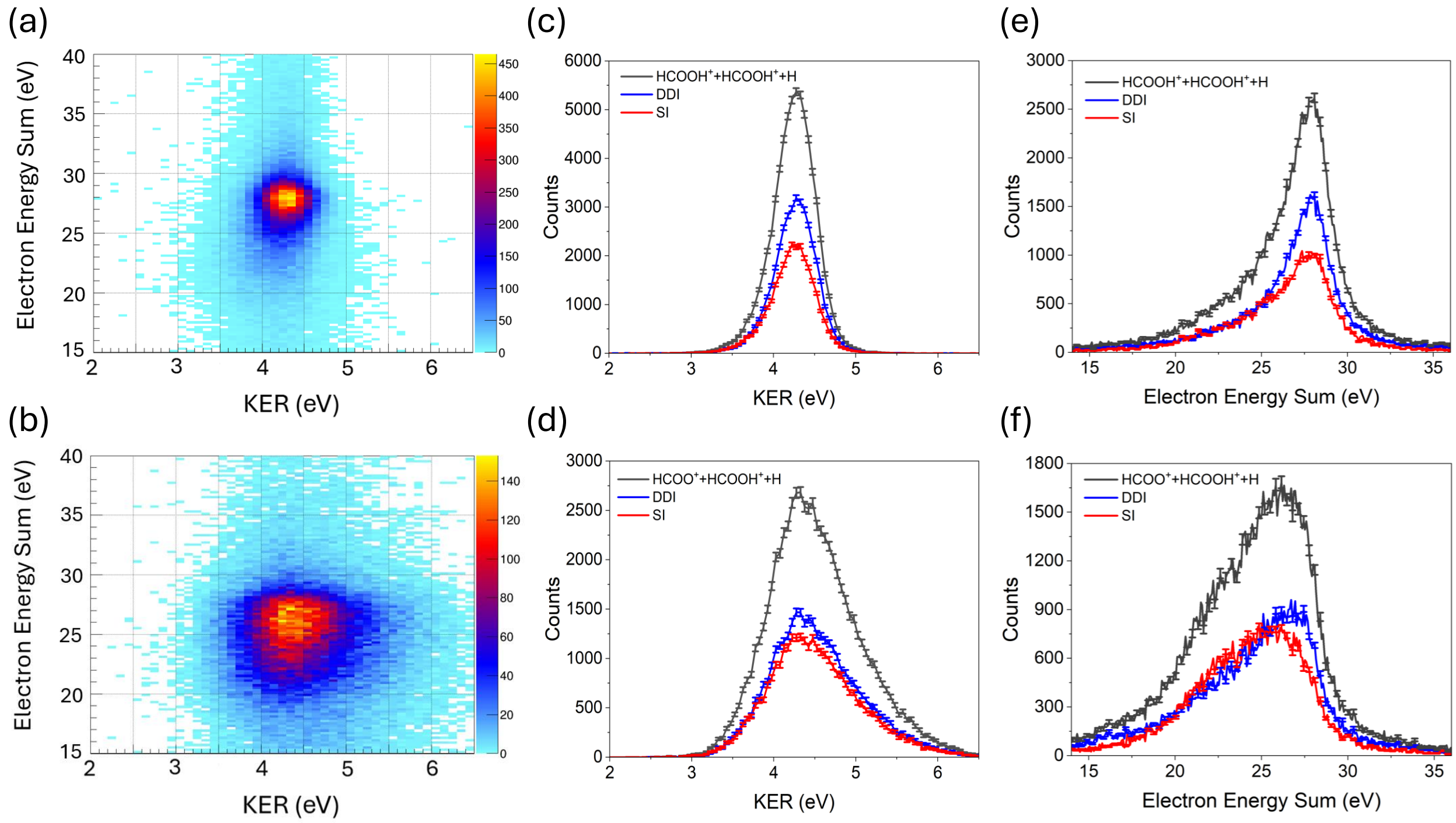

Figure S3: Top row: Symmetric 2-body dimer fragmentation channel. Bottom row: Asymmetric 3-body dimer fragmentation channel. (a) + (b): Electron energy sum vs. KER. (c) + (d) KER distributions for (black) all events leading to the 2- and 3-body fragmentation channels, respectively, and for the (blue) DDI and (red) SI events. The heatmap color scales are linear. (e) + (f) Electron energy sum ($E_{e_{sum}}$) distributions for all events leading to the 2- and 3-body fragmentation channels, respectively, and for the (blue) DDI and (red) SI events. All error bars in panels (c) to (f) reflect one standard deviation of the statistical uncertainty. DDI is gated using $eE_s \in [0.2, 0.8]$, and SI is gated using $eE_s \in [0, 0.2]$ and [0.8, 1.0], respectively. See details in Section 2.3.

2.3 Experiment: Separation of direct double ionization (DDI) and sequential ionization (SI) for the symmetric 2-body and asymmetric 3-body dimer fragmentation channels

The DDI and SI processes are distinguished based on the electron energy sharing ratio eE_s, defined in Eq. (S15), where E_{e_1} and E_{e_2} represent the energies of the two correlated electrons detected in each event:

$$eE_s = \frac{E_{e_1}}{E_{e_1} + E_{e_2}} \tag{S15}$$

Although this metric helps to separate the two ionization mechanisms, a complete dis-

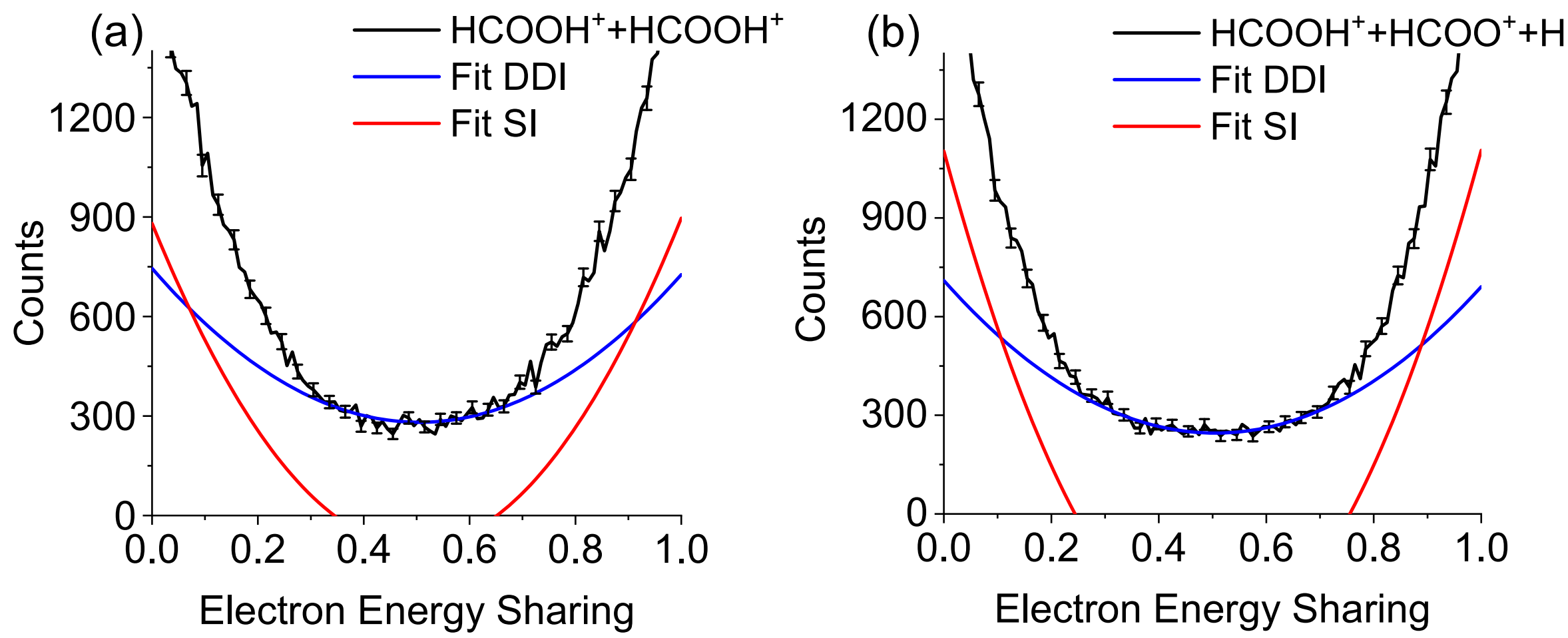

Figure S4: The electron energy sharing eE_s for (a) the symmetric $HCOOH^+ + HCOOH^+$ and (b) the asymmetric $HCOOH^+ + HCOO^+ + H$ (b) breakup channels. All error bars reflect one standard deviation of the statistical uncertainty. The polynomial fitted contributions for both DDI and SI process are shown as blue and red lines, respectively.

entanglement is not feasible due to significant overlap in eE_s, particularly near the lowest ($eE_s \leq 0.3$) and highest ($eE_s \geq 0.7$) values, as well as in other differential observables. To estimate the relative contributions of DDI and SI, we fit distinct regions of the energy-sharing distribution. The central ranges $eE_s \in [0.2, 0.8]$ are dominated by DDI events and are modeled using a second-order polynomial fit (blue curves); see Fig. S4. For an electron sum energy of $E_{e_{sum}} = 27.5$ eV, the quadratic fit closely reproduces the exponential behavior observed for eE_s in the photo double ionization of He (see Fig. 4 and Eq. 12 in Ref.[4]). Importantly, our fitting procedure does not rely on the target-specific shape parameter k, which is known for He but not for formic acid dimers, and instead is guided by our experimental results. After subtracting the DDI fit from the full distribution, the remaining data in the peripheral regions $eE_s \in$ [0,0.2] and [0.8,1.0], where SI is more prominent, are fitted with a separate second-order polynomial (red curves). These fits are shown in Fig. S4 for both (a) the symmetric and (b) the asymmetric fragmentation channels of the formic acid dimer (FAD). The integrals of each fit were used to derive the branching ratios of the two processes that are reported in Tab. 1 in the main text.

The DDI component dominates the contributions between $E_{e_{sum}} = 27$–30 eV for the symmetric channel and 25–29 eV for the asymmetric channel in Fig. S5 (indicated by the regions between the black diagonal lines with -1 slope), providing an accurate estimate of the DDI electron energy sum. To resolve the overlap between the DDI and SI contributions, notably the regions around $eE_s \in$ [0,0.3] and [0.7,1] in Fig. S4, we implemented the following procedure:

1. Isolate DDI Electron Energy Sum: Gating the data on near-equal energy sharing $eE_s \in [0.4, 0.6]$ within the diagonal boundaries in Fig. S5, where the DDI contribution

is most distinct.

2. Extract Parameters: Obtain the more accurate DDI electron energy sum peak position from Step 1 and retrieve the branching ratios of DDI and SI from the fit in Fig. S4.

3. Resolve SI: Fit the total electron energy sum with a two-peak model constrained by the parameters from Step 2 to isolate the SI electron energy sum.

A similar procedure was employed to identify the photoelectron energy peaks for both the DDI and SI contributions.

2.4 Experiment: Separation of autoionization from ICD for the symmetric 2-body and asymmetric 3-body dimer fragmentation channels

Building on the observed fragmentation channels, the role of intramolecular autoionization within the formic acid monomer becomes particularly relevant when disentangling the competing pathways in the dimer. In this scenario, initial photoionization of one monomer may produce an excited intermediate HCOOH* which releases a low-energy electron via autoionization. This additional electron contributes to the overall spectrum but does not require direct energy transfer from the partner monomer. Instead, the fragmentation pattern of the

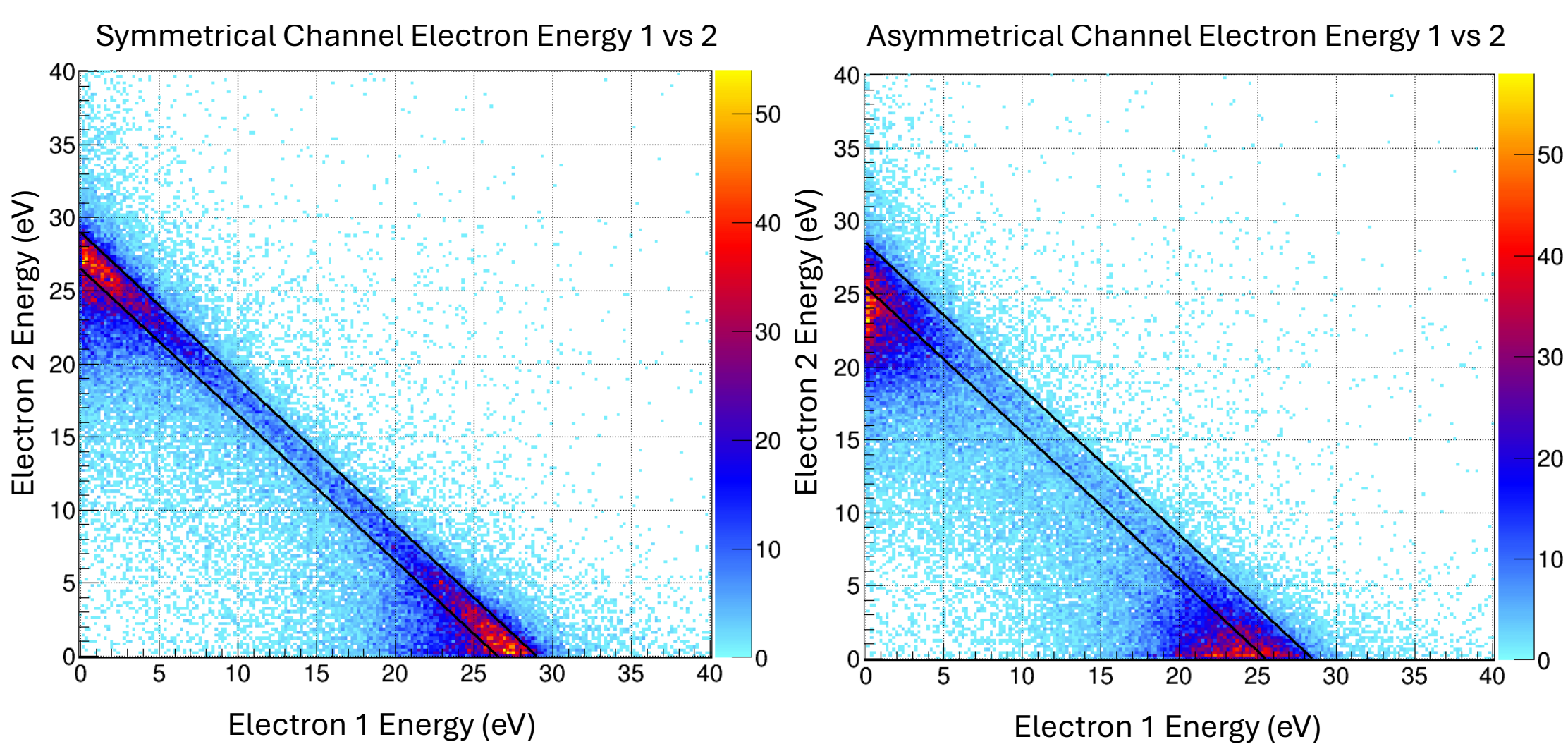

Figure S5: Two-dimensional electron energy correlation maps (electron 1 vs. electron 2) for the symmetric and asymmetric fragmentation channels. The electron energy sums ($E_{e_{sum}}$) are represented by the diagonal distributions with -1 slope between the black lines. The off-diagonal feature, highlighted by the black border, corresponds to the DDI contribution. The two distinct intensity islands indicate a mixed region where both DDI and SI processes contribute. The heatmap color scales are linear.

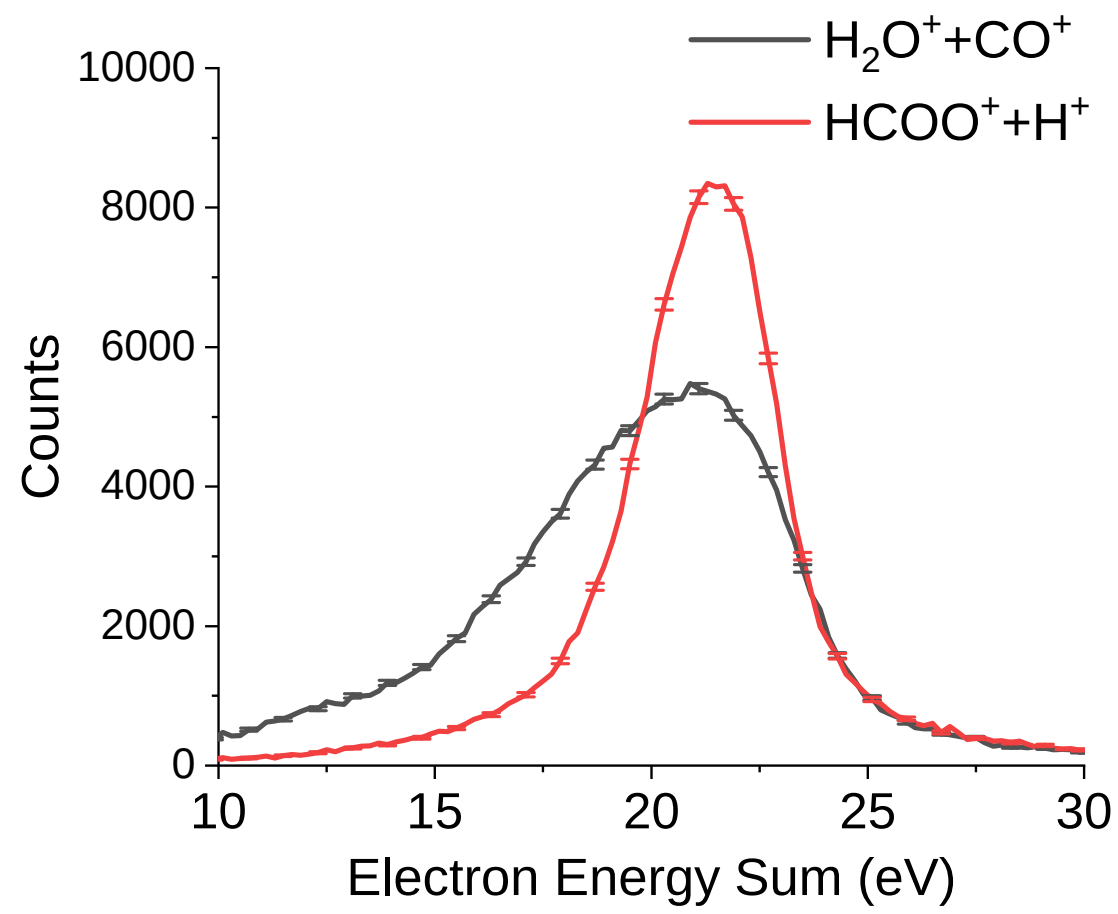

Figure S6: The electron energy sums $E_{e_{sum}}$ for the monomer breakup H_2O^+ + CO^+ (black) and $HCOO^+$ + H^+ (red). All error bars reflect one standard deviation of the statistical uncertainty.

dimer reflects a combination of the direct intermolecular ionization–decay channels and such localized monomer-centered autoionization events. Distinguishing between these contributions requires careful comparison of the dimer breakup to the reference monomer spectrum, especially in channels where the fragmentation proceeds through one intact $HCOOH^+$ ion accompanied by smaller ionic products. The electron energy sum $E_{e_{sum}}$ of two different monomer fragmentation pathways are shown in Fig. S6.

A useful diagnostic for separating DDI and SI mechanisms is the electron energy sharing ratio between the two emitted electrons (see Fig. S7). Similar to the above intermolecular Coulombic decay (ICD) electron energy sharing plot (Fig. S4), when autoionization proceeds within the monomer, the electron pair tends to exhibit asymmetric energy partitioning, with one higher energy photoelectron and one lower-energy autoionization electron. By correlating this asymmetry with the specific ionic breakup channel, one can identify the signature of monomer-centered decay processes, such as autoionization. Thus, the combined analysis of electron energy sharing and channel specific fragmentation offers a pathway to quantitatively assess the interplay between localized autoionization and intermolecular relaxation in the photoionized formic acid dimer.

2.5 Experiment: Electron angular distributions for the symmetric 2-body and asymmetric 3-body dimer fragmentation channels

To further probe the underlying ionization dynamics, we analyzed the angular distributions of the emitted electrons. During the DDI process, simultaneous electron emission is most likely facilitated via a knock-out mechanism, showing a preference for back-to-back emission,

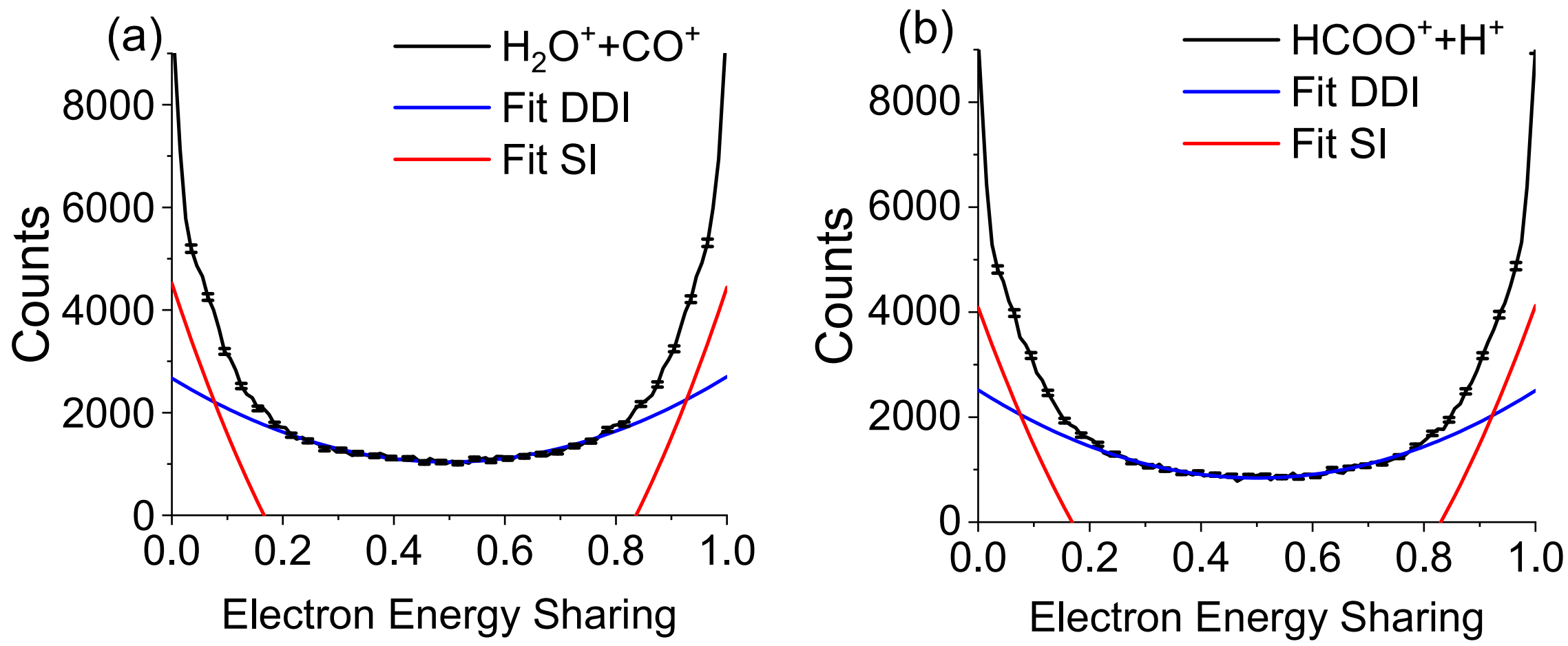

Figure S7: The electron energy sharing eE_s for monomer breakup - (a) H_2O^++CO^+ and (b) $HCOO^+$ + H^+. All error bars reflect one standard deviation of the statistical uncertainty. The polynomial fitted contributions for both DDI and SI process are shown as blue and red lines, respectively.

with the second electron preferentially ejected opposite to the first - indicative of the Coulomb repulsion between the two negatively charged particles. This manifests as a deep minimum centered at 0° in the angular correlation for DDI [Fig. S8(a, b), black dots]. In contrast, SI exhibits minimal angular correlation, as the electrons are emitted independently [Fig. S8(a, b), red diamonds].

The electron angular distributions relative to the light polarization direction confirm these interpretations. The e_P in SI follows a p-wave-like emission pattern [Fig. S8(c, d), black dots], indicative of the photoionization step. Meanwhile, the e_{LEE} distribution is isotropic (red diamonds), suggesting an unimolecular emission in a subsequent step that is independent of the light polarization. The absence of electron-electron correlation and electron-light polarization angular dependence of the e_{LEE} in SI supports a sequential mechanism, excluding electron knock-out.

Using 23 eV as the cutoff energy between the pure ICD regime and the regime in which both ICD and AI are energetically feasible, we separated the electron angular distribution into mixed and ICD-only contributions [Fig. S8(e-h)]. The electron angular distributions were fitted using the dipole differential cross-section:

$$\frac{\partial\sigma(\hbar\nu,\theta)}{\partial\omega} = \frac{\sigma}{4\pi}[1+\beta\, P_2(\cos\theta)], \tag{S16}$$

where θ is the angle between electron emission direction and the light polarization, σ is the total cross section, and P_2 is the second Legendre polynomial:

$$P_2(\cos\theta) = \frac{1}{2}(3\cos^2\theta - 1), \tag{S17}$$

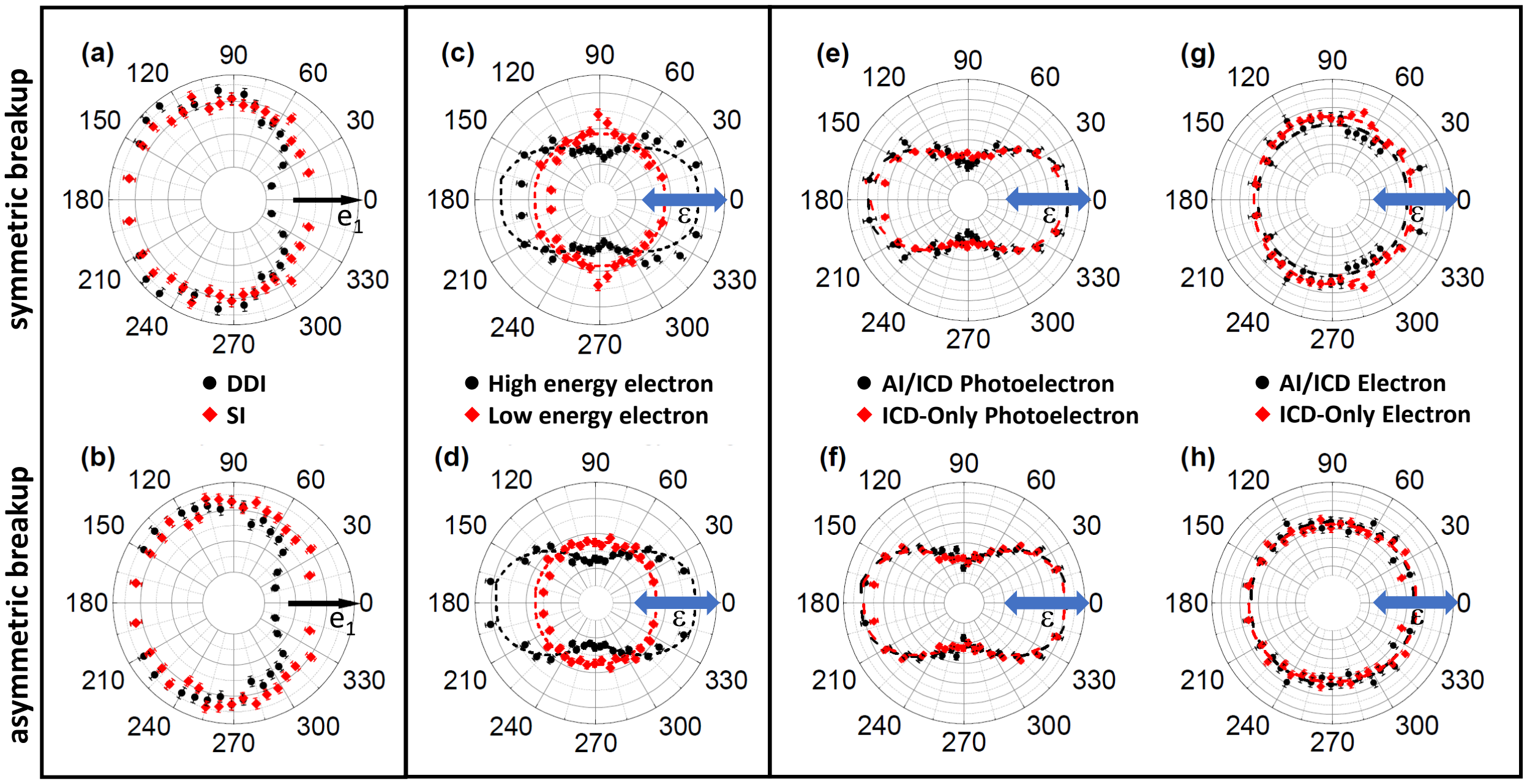

Figure S8: Electron angular distributions for the symmetric (top) and asymmetric (bottom) fragmentation channels. (a, b) SI (red) and DDI (black) electron angular distributions averaged over all polarization directions for symmetric (top) and asymmetric (bottom) $(HCOOH)_2^{2+}$ breakup, respectively, with one electron (e_1) fixed at 0° angle (single black arrow). (c-h) SI pathway only. (c, d) Angular distributions relative to the photon polarization for high- and low-energy electrons in the symmetric (top) and asymmetric (bottom) fragmentation channels. (e, f) Using 23 eV as a photoelectron cutoff energy [see Fig. 2(d) and (f)], separated mixed AI/ICD (black) and ICD-only (red) high-energy photoelectron angular distributions for the symmetric (top) and asymmetric (bottom) channels. (g, h) Corresponding low-energy mixed AI/ICD (black) and ICD-only (red) electron angular distributions. The blue double sided arrows indicate the light polarization (ϵ) direction. All error bars reflect one standard deviation of the statistical uncertainty.

with β representing the photo-excitation energy-dependent anisotropy parameter.[5,6] Since the total cross-section was not measured in this experiment, σ was fitted to the normalized event counts and is not presented here. However, these fit parameters were used to calculate the relative contributions of the AI/ICD and mixed/ICD-Only mechanisms in the symmetric and asymmetric fragmentation channels. The fitted β values and relative yields with uncertainties are summarized in Table 2 in the main text. All channels exhibit moderately positive photoelectron anisotropy parameters ($\beta_{e_p} \approx 0.6$–0.7), indicating a preferred photoelectron emission along the polarization axis. This is consistent with the outgoing electrons primarily emitted from σ orbitals, producing photoelectron angular distributions dominated by p-wave continuum contributions.

The electrons may also originate from hybridized orbitals, producing a coherent superposition of two partial waves, such as s-p π bonding orbital, thus resulting in a more p- or

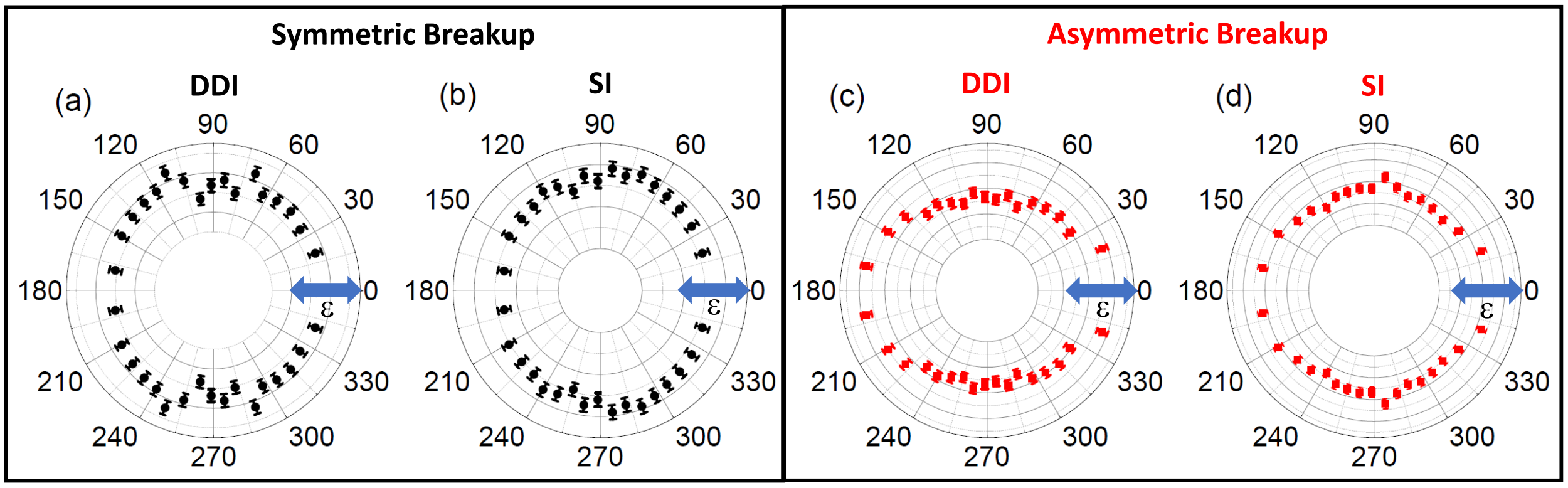

Figure S9: The angular distributions of the recoil axes between the ionic fragments relative to the polarization direction (horizontal, as indicated by the blue double arrow) for (a) DDI in the $HCOOH^+$ + $HCOOH^+$ channel, (b) SI in the $HCOOH^+$ + $HCOOH^+$ channel, (c) DDI in the $HCOOH^+$ + $HCOO^+$ + H breakup, and (D) SI in the $HCOOH^+$ + $HCOO^+$ + H breakup. All error bars reflect one standard deviation of the statistical uncertainty.

d-shape with less defined nodes for the photoelectron angular distribution. We see that β_{e_p} for the mixed contribution compared to the symmetric fragmentation channel is somewhat higher than the three other pathways listed in Table 2 in the main text. As such, the differences in β between Sym/Asym and mixed/ICD-Only channels are small ($\Delta\beta < 0.12$), suggesting that the details of the decay mechanism may have only a weak influence on the angular momentum composition of the emitted electron; a detailed comparison with photoionization calculations, which are beyond the scope of this investigation, would shine more light on these small differences.

Unlike the photoelectrons, the e_{LEE} angular distributions are nearly circular in shape, demonstrating that the emission of the slow secondary electrons through autoionization (AI) or ICD are not significantly influenced by the preceding photoionization process [Fig. S8(g, h)]. Correspondingly, the fitted $\beta_{e_{LEE}}$ parameters lie between -0.03 and 0.05 (Table 2 in the main text) and confirm that these secondary electrons exhibit negligible dipole character.

2.6 Experiment: Ion angular distributions and correlations for the symmetric 2-body and asymmetric 3-body dimer fragmentation channels

We examined the angular distributions of the recoil axis relative to the photon polarization axis for both the symmetric two-body and asymmetric three-body breakup channels. For the symmetric breakup shown in Fig. S9(a) and (b), both DDI and SI exhibit isotropic angular distributions, indicating no preferred recoil direction. This likely reflects the four-fold oxygen symmetry of $(HCOOH)_2$, which cannot be distinguished experimentally.

For the asymmetric breakup, the pairwise momentum correlations shown in Fig. S10

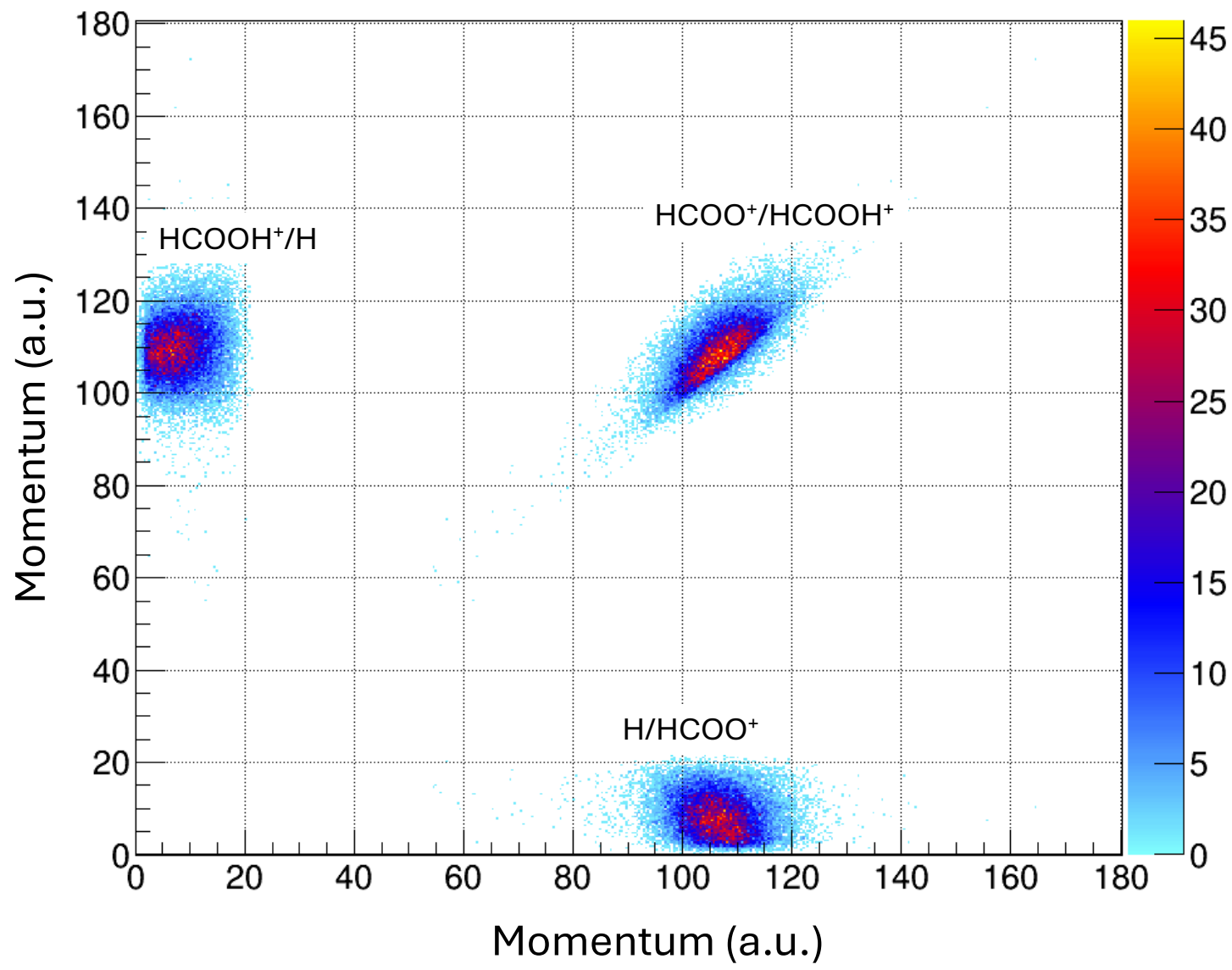

Figure S10: The fragment pair momentum-correlation diagram for the asymmetrical breakup channel $HCOOH^+$ + $HCOO^+$ + H in the lab frame. The labels indicate the correlation islands for each fragment pair combination of this three-body breakup. The neutral H fragment carries on average ∼ 7.0 a.u., while the heavy fragment ions $HCOOH^+$ and $HCOO^+$ received ∼ 109.6 a.u. and ∼ 106.6 a.u., respectively. The -1 slope of the H/$HCOO^+$ island points to a sequential breakup of $HCOOH^+$ into $HCOO^+$ + H. The heatmap color scale is linear.

reveal that the neutral H fragment carries less than one tenth of the momentum of the ionic fragments and therefore acts largely as a spectator. Consequently, the axis connecting the ionic (heavy) fragments provides a good approximation of the dimer orientation at breakup. In this channel, the DDI events display an anisotropic, elliptical distribution aligned with the polarization axis [see Fig. S9(c)], suggesting a prompt fragmentation along the molecular bonds such as C–H or O–H. In contrast, the SI events remain nearly isotropic [see Fig. S9(d)], with only slight broadening, which can be attributed to the neglected H-fragment momentum. This behavior is consistent with a slower, stepwise dissociation involving an intermediate $HCOOH^*$ state that can rotate prior to fragmentation into $HCOO^+$ + H.

2.7 Experiment: Details of the experimental setup

In the ion-electron 3D momentum imaging spectrometer, which resides in the reaction microscope endstation, the ion fragments were guided by two separate DC electric fields (acceleration region - 47 mm & 11.2 V/cm, booster region - 22 mm & 600 V/cm) toward a position- and time-sensitive detector, consisting of a 120 mm (MCP) chevron stack with a two-layer delay-line quad-anode. The electrons were collected by the electron-arm (acceleration region - 70 mm & 11.2 V/cm, drift region - 138 mm & 0 V/cm) with a similar position- and

time-sensitive detector, consisting of an 80 mm MCP with a three-layer delay-line readout (hex-anode layout). A DC magnetic field of 9.8 G with a direction coaxial to the time-of-flight axis of the spectrometer and established by a set of Helmholtz coils was employed, in order to guide electrons with up to 25 eV to the detector with 4π solid angle.

The photon beam was horizontally polarized and the polarization was oriented parallel to the Time-of-Flight (TOF) axis of the COLTRIMS spectrometer. The Advanced Light Source (ALS) was operated in two-bunch mode, and the monochromator of the beamline was set to an energy of 56 eV with a resolution below 100 meV. Formic acid dimers were generated in a supersonic expansion of formic acid gas through a 50 micron nozzle with a backing pressure of around 2-3 bar, controlled by the temperatures of the gas jet system. The temperatures of the reservoir holding the HCOOH liquid and the gas lines are kept at 45° C, and the nozzle temperature was set to 50° C. This resulted in the following pressure levels of the two-stage supersonic gas jet: The first stage of the jet is maintained around 1.0 x 10^{-4} torr, the second stage is maintained around 1.0 x 10^{-6} torr, and the photoionization chamber pressure is maintained around 8.0 x 10^{-9} torr. During the experiment, two electrons and two ionic fragments were collected in coincidence on a event-by-event basis. In case of the three-body breakup of the target gas, the 3-D momentum of the neutral fragment was derived using momentum conservation.

3.1 Theory: Details of static electronic structure calculation

We performed a set of static electronic structure calculations to analyze the energetics of the mono- and dicationic states of $(HCOOH)_2$. The geometry corresponds to the minimum energy structure at the CCSD(T)/aug-cc-pVTZ level of theory.[7] The first 40 singlet and triplet states of the dication of the dimer and the ground state dicationic energy of the monomer were calculated at the EOM-DIP-CCSD/cc-pVDZ[8] level of theory as implemented in QChem 6.3.1.[9] These energies were subtracted from the photon energy and are plotted as the spectrum in Fig. 2(e) in the main text with a 0.20 eV Gaussian broadening and as the vertical lines in Fig. 2(f). The value of the broadening was chosen to match previous work.[10] The pole strengths of the mono-cation states of $(HCOOH)_2$ were calculated at the ADC(3)/cc-pVDZ level of theory as implemented in the PySCF 2.10.0 quantum chemistry package.[11] The ADC(3) spectrum was separated into contributions from the specific O 2s-like molecular orbital (MO)s using the MO components of the Dyson orbitals and then convolved with Gaussian functions with a full-width at half maximum of 0.20 eV to introduce broadening into the spectra.[10] These spectra were subtracted from the 56 eV photon energy and were plotted as the teal and orange lines in Fig. 2(f) in the main text.

3.2 Theory: Choice of range-separation parameter

In this work, we use the tuned long-range corrected LC-PBE* functional. We use a previously developed procedure to obtain a good choice of the range-separation parameter μ by minimizing the following function[12]

$$J(\mu) = |IP_{\mathrm{SCF}}(\mu) - IP_{\mathrm{Koopman}}(\mu)| = |E^{\mathrm{cation}}_{SCF}(\mu) - E^{\mathrm{neutral}}_{\mathrm{SCF}}(\mu) + \varepsilon^{\mathrm{neutral}}_{\mathrm{HOMO}}(\mu)|, \tag{S18}$$

The subscript SCF corresponds to a ground-state DFT energy and $\varepsilon^{\mathrm{neutral}}_{\mathrm{HOMO}}(\mu)$ is the energy of the HOMO from the neutral ground-state DFT calculation. The range-separation parameter is chosen based on calculations on a single isolated monomer and is provided in Table S1.

Table S1: The values, in atomic units, of the range-separation parameter, μ, and the CAP parameters, ε_0, ξ, and γ_0.

Parameter	Value
μ	0.37
ε_0	0.049
ξ	0.5
γ_0	1.0

3.3 Theory: Choice of parameters in the complex absorbing potential

The damping matrix, $\mathbf{\Gamma}^{'}(t)$, is obtained from a time-independent diagonal matrix, $\mathbf{D}$, such that[12–14]

$$\mathbf{\Gamma}^{'}(t) = \mathbf{C}^{'}(t)\mathbf{D}\mathbf{C}^{'}(t)^{\dagger}, \tag{S19}$$

where $\mathbf{C}'(t)$ corresponds to the eigenvectors of the time-dependent Fock matrix in the orthogonalized AO basis, $\mathbf{F}^{'}(\mathrm{t})$. The diagonal matrix $\mathbf{D}$ contains exponentially increasing elements, γ_i, given by

$$\gamma_i = \begin{cases} 0, & \text{if } \varepsilon_i - \varepsilon_0 < 0 \\ \gamma_0 e^{-\xi(\varepsilon_i - \varepsilon_0)} - 1, & \text{if } \varepsilon_i - \varepsilon_0 > 0 \end{cases} \tag{S20}$$

We follow the same procedure from our previous work to obtain the values of γ_0, ε_0, and ξ, with the final values provided in Table S1.[13] Specifically, γ_0 is chosen to be 1 Ha, and the value of γ_i is capped to a maximum value of 100 Ha. The value of ξ is chosen to be as large as possible, but not so large as to introduce artificial over-ionization of the system.

The value of the cut-off energy, ε_0, is chosen to partition the virtual orbitals into a set of bound and a set of continuum orbitals based on their energy in comparison to the following

estimate of the electron affinity:[12]

$$EA_1 = E^{anion} - E^{neutral} \tag{S21}$$
$$EA_k \simeq EA_1 + \nu_{k-1} \qquad k = 2, 3, \ldots \tag{S22}$$

where ν_k corresponds to the kth TDDFT excitation of the anion. For a given value of k, EA_k and EA_{k+1} will switch from negative to positive. The value of ε_0 is chosen to match the interpolated value between ν_k and ν_{k+1}, such that the electron affinity is equal to zero. This procedure is performed based on calculations of a single isolated monomer.

3.4 Theory: Definition of the localized monomer basis

The localized monomer basis is defined as the set of MOs obtained from ground-state DFT calculations on each monomer in isolation. Thus, the population of the localized MOs are obtained through a rotation of the time-dependent 1-electron reduced density matrix (1RDM) in the non-orthogonal AO basis, $\mathbf{P}$, into the localized monomer basis, $\tilde{\mathbf{P}}$, such that

$$\tilde{\mathbf{P}} = \tilde{\mathbf{C}}^{\dagger}\mathbf{SPS}\tilde{\mathbf{C}}. \tag{S23}$$

The matrix $\mathbf{S}$ is the overlap matrix of the AOs, and $\tilde{\mathbf{C}}$ contains the combined MOs of each isolated monomer after an orthogonalization procedure as performed through the TiDES "NOSCF" routine.[15]

3.5 Theory: Initialization of the hole

Fig. S11 shows that the ground-state MOs of the full FAD are delocalized across both monomers. Consequently, we rotate the ground-state DFT density into the localized monomer basis using Eq. (S23). After rotation, the diagonal element that corresponds to either the ionized 4a' or 5a' MO is manually changed to 1, before rotating back into the AO basis using

$$\mathbf{P} = \tilde{\mathbf{C}}\tilde{\mathbf{P}}\tilde{\mathbf{C}}^{\dagger}. \tag{S24}$$

This 1RDM is then used as the starting point for the real-time time-dependent density functional theory (RT-TDDFT) calculation.

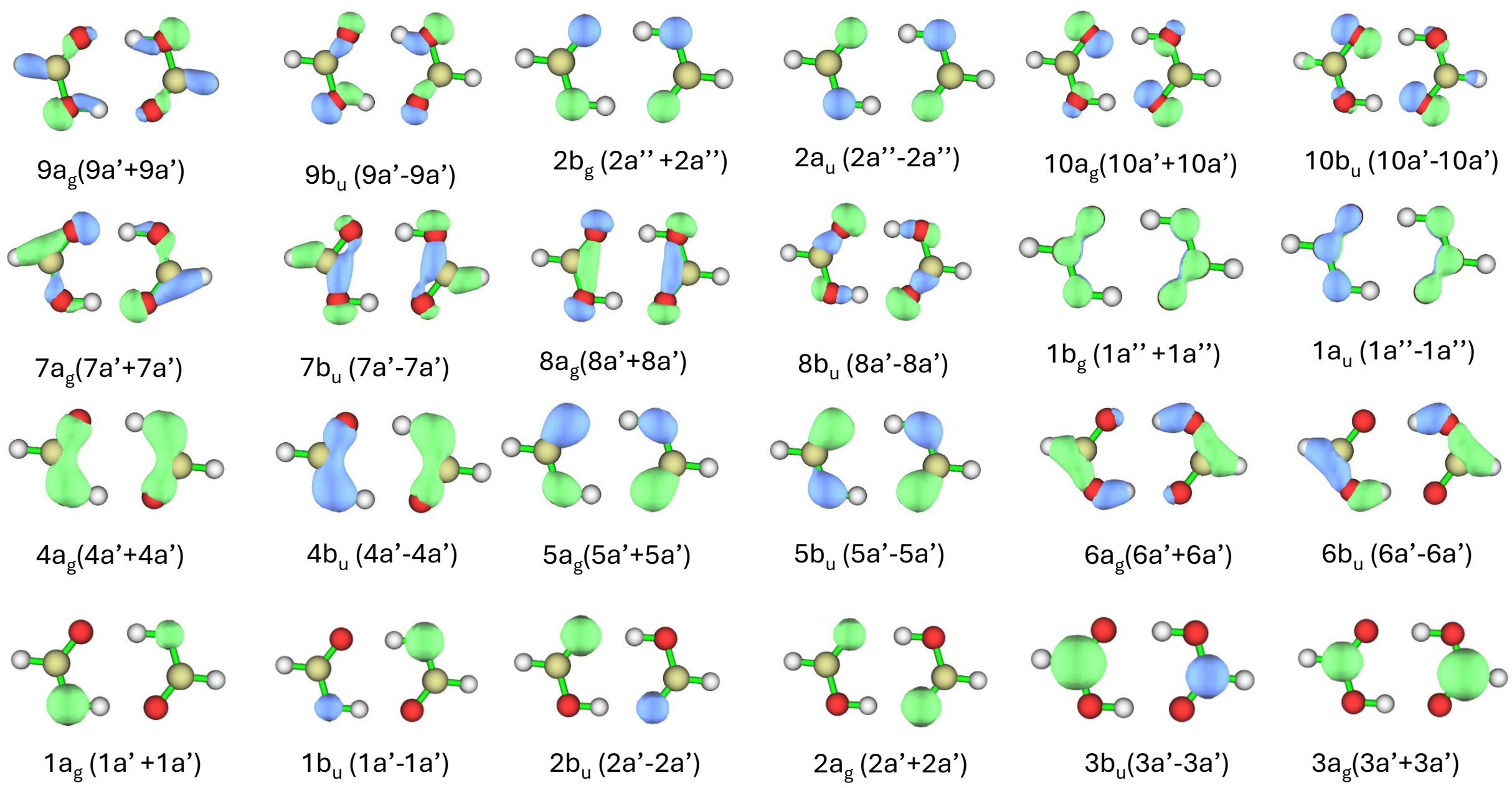

Figure S11: Images of the delocalized MOs obtained from a ground-state DFT calculation of the full FAD plotted using Multiwfn.[16] The MOs are ordered in increasing energy from left to right and from top down. The MO labeling includes the symmetry of the delocalized MO as well as the two localized monomer MOs that mainly mix to form the corresponding delocalized MO. The latter is denoted in parentheses.

3.6 Theory: Definition of the charge loss

The charge loss on monomer A is defined as

$$\text{charge loss on } A = N^A_{neutral} - \sum_{i=1\in A}^{HOMO} \tilde{P}_{ii}, \tag{S25}$$

where the sum runs over the MOs only associated with monomer A. As in previous work,[13] the sum is truncated at the HOMO associated with the original ground-state of each monomer, and $N^A_{neutral}$ corresponds to the total number of electrons on neutral monomer A.

3.7 Theory: Definition of the real-time real-space hole density

The real-time real-space hole density is defined as

$$\rho(\mathbf{r},t) = \sum_{i=1}^{HOMO} (2 - \tilde{P}_{ii}(t))|\tilde{\phi}_i(\mathbf{r})|^2, \tag{S26}$$

where $\tilde{P}_{ii}(t)$ is the occupation number of the i-th orbital at time t, and $\tilde{\phi}_i$ is the wavefunction of the i-th orbital in the localized monomer basis. Analogous results are obtained when Eq. (S26) is defined in the fully delocalized MO basis.

3.8 Theory: Additional MO Images

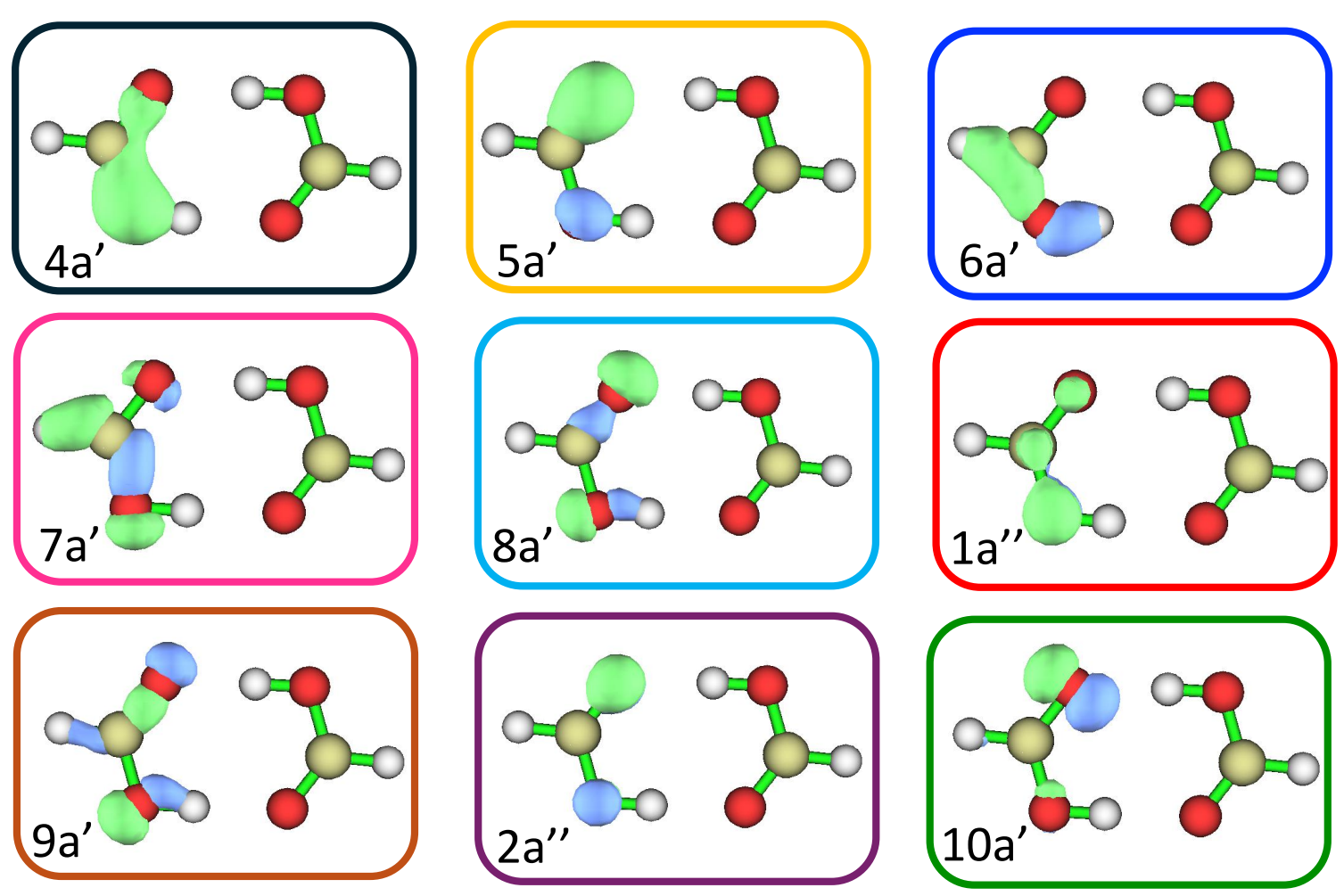

Figure S12: Images of the localized inner-shell and valence MOs on monomer-B plotted using Multiwfn.[16] These are the symmetric analogies of the monomer-A MOs presented in Fig. 4 of the main text.

References

(1) Barik, S.; Livshits, E.; Baer, R.; Strasser, D. Ultrafast and Ultraslow Proton-Transfer Dynamics Induced by Formic Acid Dimer Ionization. *The Journal of Physical Chemistry A* **2025**, *129*, 7768–7774.

(2) Barik, S.; Livshits, E.; Baer, R.; Strasser, D. Non-adiabatic origin of roaming OH dynamics in the formic acid dimer dication. *Physical Chemistry Chemical Physics* **2026**, *28*, 12952–12960.

(3) Hoshina, K.; Hagihara, H.; Tsuge, M. Double Ionization and Coulomb Explosion of the Formic Acid Dimer by Intense Near-Infrared Femtosecond Laser Pulses. *The Journal of Physical Chemistry A* **2012**, *116*, 826–831.

(4) Andersson, J.; Zagorodskikh, S.; Roos, A. H.; Talaee, O.; Squibb, R. J.; Koulentianos, D.; Wallner, M.; Zhaunerchyk, V.; Singh, R.; Eland, J. H. D.; Rost, J. M.; Feifel, R. Parametrization of energy sharing distributions in direct double photoionization of He. *Scientific Reports* **2019**, *9*, 17883.

(5) Havermeier, T. et al. Angular distributions of photoelectrons and interatomic-Coulombic-decay electrons from helium dimers: Strong dependence on the internuclear distance. *Physical Review A* **2010**, *82*, 063405.

(6) Mhamdi, A. et al. Resonant interatomic Coulombic decay in HeNe: Electron angular emission distributions. *Physical Review A* **2018**, *97*, 053407.

(7) Kalescky, R.; Kraka, E.; Cremer, D. Local vibrational modes of the formic acid dimer – the strength of the double hydrogen bond. *Molecular Physics* **2013**, *111*, 1497–1510.

(8) Kuś, T.; Krylov, A. I. De-perturbative corrections for charge-stabilized double ionization potential equation-of-motion coupled-cluster method. *The Journal of Chemical Physics* **2012**, *136*, 244109.

(9) Epifanovsky, E. et al. Software for the frontiers of quantum chemistry: An overview of developments in the Q-Chem 5 package. *The Journal of Chemical Physics* **2021**, *155*, 084801.

(10) Zhou, J.; Jia, S.; Skitnevskaya, A. D.; Wang, E.; Hähnel, T.; Grigoricheva, E. K.; Xue, X.; Li, J.-X.; Kuleff, A. I.; Dorn, A.; Ren, X. Concerted Double Hydrogen-Bond Breaking by Intermolecular Coulombic Decay in the Formic Acid Dimer. *The Journal of Physical Chemistry Letters* **2022**, *13*, 4272–4279.

(11) Sun, Q. et al. Recent developments in the PySCF program package. *The Journal of Chemical Physics* **2020**, *153*, 024109.

(12) Fernando, R. G.; Balhoff, M. C.; Lopata, K. X-ray Absorption in Insulators with Non-Hermitian Real-Time Time-Dependent Density Functional Theory. *Journal of Chemical Theory and Computation* **2015**, *11*, 646–654.

(13) Wang, Y.-S.; Zhong Manis, J. X.; Rohan, M. C.; Orlando, T. M.; Kretchmer, J. S. Modeling Intermolecular Coulombic Decay with Non-Hermitian Real-Time Time-Dependent Density Functional Theory. *The Journal of Physical Chemistry Letters* **2024**, *15*, 7806–7813.

(14) Lopata, K.; Govind, N. Near and Above Ionization Electronic Excitations with Non-Hermitian Real-Time Time-Dependent Density Functional Theory. *Journal of Chemical Theory and Computation* **2013**, *9*, 4939–4946.

(15) Rohan, M. C.; Suarez, V. A.; Clothier, M.; Kretchmer, J. S. TiDES: A time-dependent electronic structure code for real-time electron and spin dynamics. *The Journal of Chemical Physics* **2026**, *164*, 234117.

(16) Lu, T.; Chen, F. Multiwfn: A multifunctional wavefunction analyzer. *Journal of Computational Chemistry* **2012**, *33*, 580–592.
